# Pharmacological Management of Neurogenic Bowel Dysfunction after Spinal Cord Injury and Multiple Sclerosis: A Systematic Review and Clinical Implications

**DOI:** 10.3390/jcm10040882

**Published:** 2021-02-22

**Authors:** Jeffery S Johns, Klaus Krogh, Karen Ethans, Joanne Chi, Matthew Querée, Janice J Eng, Spinal Cord Injury Research Evidence Team

**Affiliations:** 1 Department of Physical Medicine and Rehabilitation, Vanderbilt University Medical Center, Nashville, TN 37232, USA; 2 Department of Hepatology and Gastroenterology, Aarhus University Hospital, 8200 Aarhus, Denmark; klaus.krogh@clin.au.dk; 3 Physical Medicine and Rehabilitation, University of Manitoba, Winnipeg Health Sciences Centre, Winnipeg, MB R3A 1R9, Canada; kethans@hsc.mb.ca; 4 Department of Physical Therapy, University of British Columbia and Rehabilitation Research Program, GF Strong Rehab Centre, Vancouver, BC V5Z 2G9, Canada; joanne.chi.1999@gmail.com (J.C.); matthew.queree@ubc.ca (M.Q.); JANICE.ENG@UBC.CA (J.J.E.); 5 Spinal Cord Injury Research Evidence Team, Vancouver, BC V5Z 2G9, Canada

**Keywords:** spinal cord injury, multiple sclerosis, neurogenic bowel dysfunction, pharmacological, systematic review

## Abstract

Neurogenic bowel dysfunction (NBD) is a common problem for people with spinal cord injury (SCI) and multiple sclerosis (MS), which seriously impacts quality of life. Pharmacological management is an important component of conservative bowel management. The objective of this study was to first assemble a list of pharmacological agents (medications and medicated suppositories) used in current practice. Second, we systematically examined the current literature on pharmacological agents to manage neurogenic bowel dysfunction of individuals specifically with SCI or MS. We searched Medline, EMBASE and CINAHL databases up to June 2020. We used the GRADE System to provide a systematic approach for evaluating the evidence. Twenty-eight studies were included in the review. We found a stark discrepancy between the large number of agents currently prescribed and a very limited amount of literature. While there was a small amount of literature in SCI, there was little to no literature available for MS. There was low-quality evidence supporting rectal medications, which are a key component of conservative bowel care in SCI. Based on the findings of the literature and the clinical experience of the authors, we have provided clinical insights on proposed treatments and medications in the form of three case study examples on patients with SCI or MS.

## 1. Introduction

Neurogenic bowel dysfunction (NBD) is a prevalent issue for people with neurological disorders; changes in bowel motility and sphincter control can present a major problem for people with spinal cord injury (SCI) and multiple sclerosis (MS). The reported prevalence of NBD varies, with most reports of constipation occurring in the range of 30–40% of people with chronic SCI. However, some studies have found the prevalence of constipation to be closer to 80%, and upwards of 75% of individuals with SCI experience fecal incontinence [1,2]. NBD is also prevalent in people with MS. A systematic review found the prevalence of constipation to range from 18–43%, and fecal incontinence occurs in 3–51% of people with MS, based on studies with over 100 patients [3]. In the general population, constipation and fecal incontinence have been reported to be 19.7% and 4.3% respectively, in a 70,000-plus population-based sample, with increasing prevalence in older age patients [4]. Thus, it is clear that bowel dysfunction is far more prevalent in people with SCI and MS and requires special attention.

Bowel dysfunction due to SCI or MS has a substantial negative impact on quality of life [5]. Even when a bowel program is in place to effectively manage NBD, it can be onerous and time-consuming and may take up to 1–2 h per session, repeated every day or alternate days. It can interfere significantly with a person’s education, work, and social life and presents a major challenge to quality of life, independence, and community reintegration after SCI. Loss of bowel control is a source of anxiety and distress [6,7]. Treatment of bowel dysfunction rates highly for patients in both clinical and research domains of SCI and MS [8,9]. Regaining bowel function has been ranked similarly in priority to regaining walking after SCI [10].

The major symptoms of NBD are fecal incontinence and constipation. Fecal incontinence is the accidental passing of bowel movements, including solid stools, liquid stools, or mucus. This often occurs if muscles in the rectum and anus are not functioning to store and hold back a bowel movement due to muscle injury or nervous system damage, as well as a loss of rectal sensation [11]. Constipation is defined as a reduction in the frequency of stools, but a lack of a daily bowel movement is not necessarily equivalent to constipation as some people have as few as three bowel movements per week. Symptoms of constipation could include difficulty with stool passage, infrequent bowel movements or passage of hard stools [12].

Generally, people with higher and more severe injuries tend to have more significant bowel dysfunction, particularly constipation [13]; the studies by Liu [14,15] found that severity of NBD was significantly higher for people with higher American Spinal Cord Injury Association Impairment Scale (AIS) score classification and that people with AIS A SCI were at 12.8 times greater risk of severe NBD than those with AIS D.

There are two distinct patterns in the clinical presentation of bowel dysfunction in SCI: injury above the conus medullaris results in upper motor neuron (UMN) bowel syndrome, while injury at the conus medullaris and cauda equina results in lower motor neuron (LMN) bowel syndrome [2,16]. The upper motor neuron bowel, or hyperreflexic bowel, usually occurs with injuries above the sacral spinal cord and is characterized by loss of voluntary (cortical) control of the external anal sphincter, which remains involuntarily overactive, thereby promoting retention of stool. Transit time is prolonged throughout the colon. Fecal incontinence occurs concomitantly in many cases due to reduced or absent anorectal sensation and lack of voluntary control of the external anal sphincter muscle. Although there is the loss of supraspinal control, the nerve connections between the spinal cord and the colon remain intact; therefore, there is preserved reflex coordination and stool propulsion. Stool evacuation in these individuals occurs in response to stimulation of reflex activity, such as the presence of feces in the rectum, a suppository, enema, or digital rectal stimulation causing rectal distension.

The lower motor neuron bowel, or areflexic bowel, usually occurs with injuries at the sacral spinal cord or below and is characterized by the loss of centrally mediated (spinal cord) peristalsis and loss of reflex activity, resulting in slow stool propulsion and impaired reflex stool evacuation. Segmental colonic peristalsis occurs only due to the activity of the enteric nervous system, which is slower and less efficient without the centrally mediated peristalsis. The result is increased transit time through the distal colon and rectum with the production of drier and round-shaped stool. Lower motor neuron bowel syndrome is commonly associated with constipation. There is also a substantial risk for fecal incontinence due to the atonic external anal sphincter and lack of sensation and voluntary control over the external anal sphincter muscle.

In MS, the pattern of bowel dysfunction is similar to the pattern described for SCI. The neurological lesion is, however, less well defined in MS. The presence of bowel symptoms in MS is correlated to the expanded disability status scale [17], to the degree of spinal atrophy [18], and to disease duration, but not particularly with the type of MS [19]. The precise neuropathological mechanism in NBD and MS is not completely defined, but one study theorizes that at the cortical level, demyelination within the frontal lobe may affect a person’s voluntary control over bowel movements [20]. Regardless, it has been noted that severe constipation is often one of the first presenting symptoms of MS [21].

A regular bowel program helps to ensure that evacuation occurs regularly– facilitating continence and reducing constipation. Prevention of constipation will reduce symptoms, such as abdominal pain and bloating and minimize the development of anorectal morbidities associated with NBD, including hemorrhoids, anal fissure, rectal abscess, and rectal prolapse.

**Figure 1:**
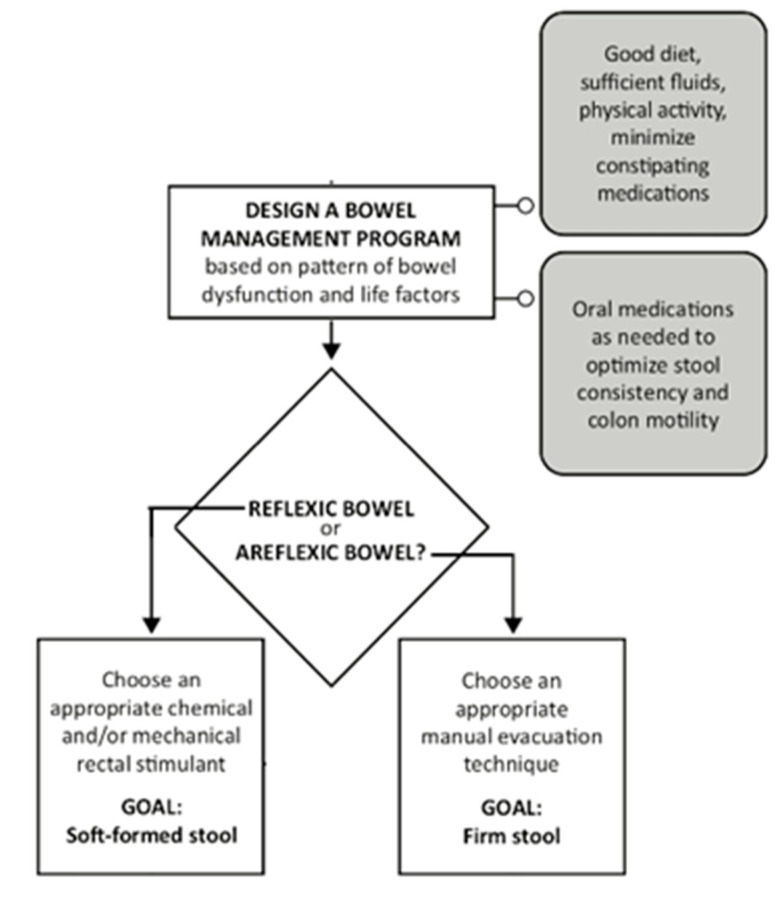
Designing a neurogenic bowel program. Reprinted (a portion of the original algorithm) from “Management of Neurogenic Bowel Dysfunction in Adults after Spinal Cord Injury: Clinical Practical Guidelines for Healthcare Providers (2020), with permission from Paralyzed Veterans of America.

A comprehensive bowel program will combine a number of interventions in an individualized routine and may include a specific diet to ensure adequate fiber and fluid, digital rectal stimulation, digital removal of stool, stimulation of the gastrocolic reflex, and use of oral or rectal (suppositories, enemas) medication. The different components of a bowel program are illustrated in Figure 1. Such a program will usually be performed on a daily or alternate day basis, depending on the needs of the individual. Undertaking physical activity, including standing and passive movements, may also help to reduce constipation. Some medications that are being used for other medical conditions or symptoms may also contribute to constipation. If these additional medications cannot be eliminated, stool softeners or oral laxatives may be used to modulate stool consistency and promote stool transit.

Neurogenic bowel guidelines [22,23] recommend that a conservative bowel program should be developed initially in the rehabilitation phase following injury and that a comprehensive evaluation of bowel function and management is undertaken at least annually. The evaluation may include a patient history (including a detailed history of current bowel routine management, stool form, continence and time spent on evacuation, diet and fluid intake, relevant medical conditions and medications, the extent of care provision and home adaptations) and a detailed physical examination (including neurological examination to determine level and completeness of SCI as well as an abdominal and rectal examination). In some centers, comprehensive assessment tools, such as the International Spinal Cord Society (ISCoS) Bowel Data Set, are used to collect this information in a standardized manner.

A recent systematic review by Musco et al. [24] assessed the literature on all NBD treatments for adults, including both pharmacological and non-pharmacological approaches. From the results of the six studies included in the section on pharmacological treatments, there were statistically significant increases in weekly bowel movements and a decrease in colonic transit time with the use of 2 mg of prucalopride among individuals with SCI. However, there were no significant improvements in the duration of bowel care or the reduction of fecal incontinence and the need for digital evacuation of stool. In addition, the review found that mechanical evacuation (tap water enema) without oral stimulant laxatives was superior in bowel control (time required for evacuation) compared to irritant and stimulant-medication groups. Furthermore, from the six studies, only three included populations of individuals with SCI and none with MS, presenting a need for further investigation and clinical insights on the effectiveness of pharmacological management in NBD among both populations.

Hence, the objective of this investigation was to first assemble a list of current pharmacological agents (medications and medicated suppositories) used in current practice through the clinical expertise of our team, which included members from the United States, Europe, and Canada. Second, we systematically examined the current literature to determine the potential in managing NBD of individuals specifically with SCI or MS. We also reviewed literature outside of our designated populations of interest and with regards to other methods of bowel management to inform our approach and help us provide guidance for healthcare professionals as to when it is appropriate and timely to prescribe medication for NBD. Based on the findings of the literature and the clinical experience of the authors, we have provided clinical insights on proposed treatments and medications in the form of three case study examples on patients with SCI or MS.

## 2. Methods

### 2.1. List of Current Pharmacological Agents

We generated a list of current pharmacological agents (medications, medicated suppositories) prescribed for adults with NBD through a combination of clinical expertise from the United States, Canada and Europe and web-based searches on the drug monographs to define generic and trade names and common side effects.

### 2.2. Literature Search and Study Selection

We searched the electronic databases Ovid MEDLINE^®^, EMBASE, and CINAHL for relevant literature dated from 1980 through June 2020, using search terms related to adult bowel dysfunction (e.g., constipation, bowel/fecal incontinence), spinal cord injury (e.g., paraplegia, tetraplegia, spinal cord injury/dysfunction), Multiple Sclerosis (or MS), and the brand names/generic names of all medications used for bowel dysfunction suggested by the author team and the university health librarian. We also identified additional studies through hand-searching the reference lists of included studies and reviews. Studies on medications for colonoscopy preparation were excluded as they do not reflect treatments for daily bowel management.

Two reviewers independently assessed titles and abstracts of citations for inclusion and the quality of the studies, with disagreements resolved by a third person. Review articles were only included if it was a systematic review. All articles were limited to English only. Animal studies and articles describing the neurophysiology of bowel were excluded. Duplicate studies were identified and removed using RefWorks management software (Ex Libris, Ann Arbor, MI, USA).

### 2.3. Inclusion Criteria

Three principles guided study inclusion: (1) studies were included if the population of interest was people with SCI or MS, (2) if they measured any outcomes related to bowel or bowel-related dysfunction (e.g., using the NBD or Wexner scores, or reporting the number of occurrences of fecal incontinence or constipation, colonic transit time, or duration/frequency of bowel movements), and (3) if the independent variable or inquiry of interest was some form of medication (e.g., prucalopride) and/or medicated suppository (e.g., bisacodyl). We endeavored to include all research designs, but qualitative studies and case reports were excluded. Results published only in abstract form or in conference proceedings *could be included* if adequate details were available for quality assessment (e.g., risk of bias) and if the area of inquiry had relatively little published information. Mixed populations were acceptable if the sample consisted of at least 20% people with SCI or MS.

### 2.4. Data Extraction and Synthesis

We extracted information from included studies and constructed evidence tables showing the study characteristics, outcomes, adverse effects, and quality ratings/risk of bias for all included studies. We presented the studies using a hierarchy of evidence approach, where the best evidence is presented first in tables and is the focus of any results, point estimates, or conclusions. If no literature was found for a commonly used medication (e.g., oral laxative), then practice guidelines or meta-analyses were sought in non-NBD populations (e.g., individuals with idiopathic chronic constipation).

### 2.5. Validity Assessment (Risk of Bias)

We used the grading of recommendations, assessment, development, and evaluations (GRADE) system to provide a systematic approach for evaluating the evidence [25]. We assessed the internal validity (risk of bias) of trials, observational studies, and systematic reviews, which include an evaluation of randomization, allocation concealment, blinding, the similarity of compared groups at baseline, loss to follow-up, and the accounting for any statistical confounds.

A study with a high attrition rate (e.g., 15% or greater) or a low response rate (lower than 50%) was automatically rated as a high risk of bias. Systematic reviews were rated on the clarity of review question, specification of inclusion and exclusion criteria, use of multiple databases for searching, sufficient detail of included studies, adequate assessment of the risk of bias of included studies, and providing an adequate summary of primary studies. Observational studies were rated on non-biased selection, loss to follow-up, pre-specification of outcomes, well-described and adequate ascertainment techniques, statistical analysis of potential confounders, and adequate duration of follow-up.

## 3. Results

### 3.1. Current Bowel Oral Medication and Medicated Suppositories

Table 1 provides an overview of current medications identified by our expert clinicians. A number of oral medications were identified. Docusate sodium is a commonly used stool softener that draws water into the stool, making it easier to pass. Osmotic softeners, such as polyethylene glycol (PEG), are laxatives that increase the moisture in the stool to make it easier to pass and are usually taken once or twice per day or as needed. Stimulant laxatives activate contractions of the intestinal wall, thereby promoting transit. Commonly used oral stimulant laxatives include bisacodyl and sennosides. Prokinetic agents stimulate the contraction of the muscle cells of the gut and promote transit. Like stimulant laxatives, prokinetic agents are medications that increase digestive tract muscle activity to move the stool through digestion. Secretory drugs increase intestinal fluids, which then accelerate intestinal transit. Narcotic antagonists are used to treating opioid-induced constipation without blocking the effect of narcotics on pain.

Medicated suppositories and enemas are also commonly prescribed for NBD. Stimulant suppositories contain medications (such as bisacodyl) that stimulate the bowel reflex. Suppositories are usually inserted 15–30 min before planned bowel emptying. The time to bowel movement is influenced by the type and route of administration. For example, oral bisacodyl may produce a bowel movement within 6–12 h, a rectal bisacodyl suppository within an hour and a rectal bisacodyl enema within 20 m. However, the medication used and even the base that the medication is dissolved in can affect how quickly the medication is absorbed. For example, bisacodyl is a water-soluble polyethylene glycol base (e.g., Magic Bullet) that allows shorter times to empty than bisacodyl in a vegetable oil base [26,27]. Lubricating suppositories contain non-medicated substances (such as glycerin), which hold water in the bowel to make the stool softer, so it is easier to expel.

### 3.2. Systematic Review

We initially found 1850 articles, and after duplicates were removed, we reviewed 1576 potentially relevant records through our searches for medications (including medicated suppositories and enemas) and NBD in SCI and MS. We assessed 62 articles for eligibility at the full-text level and ultimately included 28 studies that assessed the effects of medication on NBD in the MS (*n* = 2) and SCI population (*n* = 26).

### 3.3. Indication and Efficacy by Medication from the Systematic Review

Detailed abstraction tables are available in the online supplementary. A summary of the evidence is provided below.

### 3.4. Oral Laxatives

Oral laxatives are the first-line treatment for constipation; however, no studies were found testing them specifically in SCI and MS, so we resorted to previous reviews conducted on the effects of medications on constipation in the general population. Luthra et al. [28] conducted a network meta-analysis to compare the efficacy of different medications in people with chronic idiopathic constipation. They found 33 RCTs conducted with 17,214 patients and found that stimulant laxatives bisacodyl and sodium picosulfate was ranked first after 4 weeks, and prucalopride was ranked first after 12 weeks of treatment. Similarly, Alsalimy et al. [29] found that senna and lactulose were superior to placebo when studied in long-term care patients. Paré and Fedorak [30] reviewed the literature and found that both nonstimulant and stimulant laxatives provided better relief than a placebo, albeit with minor side effects. In another meta-analysis, Nelson et al. [31] tested the number needed to treat (NNT) chronic constipation and found that osmotic and stimulant laxatives had an NNT of 3, lubiprostone had an NNT of 4, and prucalopride and linaclotide both had an NNT of 6. Note, none of these studies examined the long-term efficacy of these medications.

Given the lack of evidence in NBD populations, the prescription of oral laxatives relies on the above evidence from the general population and expert opinion. Oral laxatives are applicable to both areflexic and reflexic bowel management. In an individual with constipation after MS and SCI, we recommend starting with a simple agent, such as magnesium hydroxide (Milk of Magnesia) or PEG, which may have fewer adverse effects. Start the night before the bowel routine (typically every other day, or 3X/week), then reassess this regimen’s effectiveness after a few weeks. It should be evaluated whether the oral medications are moving the stools toward their ideal consistency (soft, formed, bulky) and have resulted in improved evacuation. If not effective, a stimulant laxative can be tried. If the patient is in earlier stages of their injury (e.g., undergoing inpatient rehabilitation), more frequent assessments (every few days) and changes may be required.

Oral medications may address constipation but may not necessarily treat fecal incontinence. This may be due to the less predictable timing of results following oral medications. The goal of treating incontinence in NBD is to trigger a bowel evacuation at a patient-preferred time, so the movement does not occur as an unexpected or unplanned event, thus becoming incontinence. While there are no studies specifically on oral medications and fecal incontinence in the MS and SCI populations, a systematic review in adults with symptoms of fecal incontinence [32] found that medications, such as lactulose and loperamide, seemed to perform better than a placebo on measures of bowel function, such as frequency, urgency, and reduction in diarrhea, though more participants experienced adverse effects (e.g., constipation, abdominal pain, diarrhea, headache, and nausea).

### 3.5. Prokinetic Drugs

When oral laxatives are not effective, prokinetic drugs may be an alternative. Evidence for prokinetic drug studies was found for prucalopride, metoclopramide and neostigmine in SCI (1 RCT for prucalopride, 2 RCTs and one observational study for neostigmine, and two observational studies for metoclopramide). Metoclopramide stimulates the muscles of the gastrointestinal tract through dopamine and acetylcholine receptors and is approved for use to treat nausea and vomiting associated with chemotherapy, gastroesophageal reflux disease or diabetic gastroparesis. Though metoclopramide has been shown to be an effective drug to stimulate a one-time increase in gastric emptying in SCI [33], its role in ongoing neurogenic bowel management has not been established. Similarly, intravenous or intramuscular neostigmine has been shown to induce bowel evaluation in SCI but has not been tested in routine bowel management [34,35]. It is possible that metoclopramide or neostigmine may have a potential role in one-time bowel preparation procedures, such as colonoscopy in SCI.

Given that metoclopramide and neostigmine are not used for current neurogenic bowel management, the rest of this section will focus on prucalopride, a prokinetic agent that acts with high selectivity on serotonin type 4 receptors to initiate peristalsis, colonic mass movements, and facilitates defecation [36]. A systematic review of the general population found ten phase III trials that supported its efficacy and safety of prucalopride for the treatment of chronic idiopathic constipation and four phase IV trials, including one, which demonstrated efficacy over 24 months [37]. Prucalopride is recommended for idiopathic constipation if patients are not responsive to laxatives as the drug can have a high-cost [37]. Currently, tablet formulations of prucalopride have been approved in many countries and their regulating agencies, including the US Food and Drug Administration, Health Canada, and the European Medicines Agency.

A low-level of evidence, comprised of one RCT, may support the use of prucalopride to treat NBD after SCI; however, while confidence intervals were presented, no formal statistics were undertaken, which limits the interpretability of this study. Individuals who were treated with prucalopride may have experienced dose-dependent improvements in bowel movement frequency and perception of treatment efficacy. The greatest efficacy was observed at 2 mg daily dose where patients reported a 0.6 increase (95% CI 0.2 to 1.2) in weekly bowel frequency, a 73 median effectiveness rating (0 = ineffective and 100 = extremely effective), and a 38.5 h median decrease in colonic transit time [38]. Although patients receiving prucalopride perceived a higher treatment efficacy than those receiving the placebo, bowel frequency remained unchanged following a 4-week regimen of daily 1 mg prucalopride [38].

These outcomes should also be interpreted with caution as 50% of the 2 mg prucalopride group withdrew from the study, which introduces substantial bias [38]. In Krogh et al.’s study [38], adverse events were reported by 6/7 in the placebo group and by 7/8 and 6/8 in the 1 and 2 mg groups, respectively. Individuals receiving 1mg prucalopride treatment experienced the following complications more frequently than the placebo group: flatulence, bradycardia, headache, and diarrhea. Among those receiving the 2 mg prucalopride treatment, the following adverse effects were more common than in the placebo group: bradycardia, headache, abdominal pain, and diarrhea [38]. The primary medication-related reactions cited for withdrawal within the 2 mg group were headaches in combination with either abdominal pain or diarrhea [38]. The brand name Resotran monograph states hypersensitivity to Resotran, renal impairment requiring dialysis, and intestinal perforation or obstruction as contraindications [39]. Krogh et al.’s study [38] recommends starting individuals with SCI on a 1 mg daily dose before transitioning them to a 2 mg daily dose. The authors speculate that this protocol could potentially reduce dose-dependent increases in adverse events observed in the study [38].

### 3.6. Potassium Channel Blocker

Fampridine is a potassium channel blocker that can enhance synaptic transmission, and it has been approved for use to improve walking for adults with MS, but in a case series, 1 out of 23 MS participants reported improvements in urinary and fecal incontinence after six months of use [40]. Two of the four RCTs in SCI showed improvements in the number of bowel movements [41,42], but this was a secondary outcome of these studies. Currently, the mechanism by which fampridine may facilitate bowel function is unclear. While fampridine is not currently used for bowel management in current practice, the possible improvements in bowel function are intriguing; the mixed results warrant the need to study the effect of fampridine on bowel function in future studies.

### 3.7. Suppositories and Enemas

Rectal medications are typically a key component of bowel care of SCI patients with reflexic bowel or upper motor neuron lesions [23]. Rectal medications (suppositories, enemas) chemically stimulate the anal sphincter reflex to evacuate stool, and thus, the presence of an intact reflex is usually required. Suppositories are solid forms of rectal medication, while enemas are liquid, which are more difficult to insert if a patient has poor dexterity. Thus, the suppository is often first-line, especially for an individual doing their own bowel care. Rectal medications treat the dual problem of constipation and fecal incontinence. As these medications control the timing and predictability of bowel movement, they can have substantial benefits on the management of fecal incontinence. A number of cross-sectional studies demonstrate that rectal medications are used to treat more severe cases of NBD as those using rectal medications were associated with cervical injuries [6], poorer quality of life [43], extended hospitalization [44], longer bowel care [6,45], and presence of fecal incontinence [6].

Despite the common usage of suppositories, there is relatively little research on their effectiveness in SCI or MS. The small number of prospective controlled trials that have been conducted support the usage of suppositories; time to flatus, defecation sessions and total bowel care time all decreased [26,27,46]. We found only one crossover trial comparing different types of suppositories in SCI [47] that showed no significant difference in total colonic transit time between docusate sodium and benzocaine mini-enemas and mineral oil enemas, though both had a significantly shorter colonic transit time than bisacodyl or glycerin suppositories.

Of the two variations of bisacodyl suppositories, polyethylene glycol-based (PGB) bisacodyl outperformed hydrogenated vegetable-oil-based (HVB) bisacodyl across multiple outcomes and studies. Individuals receiving PGB bisacodyl had flatus 12.8–15 m after administration [26,27], 20–32 min long defecation sessions [26,27] and a total bowel care times of 43–66 min [26,27,46]. These outcomes were 44.8–58.7% faster than when HVB bisacodyl was given to the same individuals to initiate bowel care. Stiens et al. [27] attributed this difference to PGB suppositories’ more effective ability to readily dissolve from body heat, distribute bisacodyl on mucus membranes, and sustain reflex propulsion of stool. Despite the documented benefits of the PGB formulation, HVB bisacodyl suppositories are more commonly used, primarily due to the fact that the HVB version generally costs less and is easier to obtain.

When analyzed against docusate sodium and benzocaine mini-enemas in a repeated measures study with a randomized sequence of the agent, PGB bisacodyl produced comparable results [26]. The authors of this study also stated that a docusate sodium-benzocaine mini-enema was more difficult for those with limited dexterity as the serrated edge of the enema could cause anal mucosal perforation during insertion, and it required squeezing for administration [26]. In contrast, Dunn and Galka [48] demonstrated that individuals with SCI had significantly shorter evacuation times with docusate sodium-benzocaine enema than with bisacodyl. However, the type of base (HVB or PGB) of the bisacodyl suppository was not stated, which could alter these interpretations. This information was once again missing in Amir et al. [47], where bowel evacuation time was longer after bisacodyl than mineral oil enemas, docusate sodium-benzocaine enemas, or glycerin suppositories. Although in the same study, bisacodyl did reduce the difficulties of evacuations better than glycerin suppositories [47].

A bisacodyl suppository is typically used as a first-line rectal medication as it is relatively inexpensive, easier to handle than a full-sized enema, and has some evidence of its effect. The suppository is easy to insert even for individuals with impaired dexterity and does not require voluntary contraction of the external anal sphincter for retention [27]. The suppository acts as a contact irritant to enhance gastric motility, increase the fecal water content, and reduce transit-time within the large intestine [49]. The bases act as a vehicle for delivering bisacodyl, the active ingredient. Prior to insertion of a bisacodyl suppository, the rectum should be digitally checked for feces. If present, the feces should be manually evacuated. In addition, the anal canal should be lubricated with a water-based jelly. Within the SCI population, a 10 mg bisacodyl suppository is commonly prescribed as it facilitates independent care [27]. Typically, one bisacodyl suppository is used every 1–2 days for immediate effect, with a bowel movement following 15–60 min after use.

Contraindications for bisacodyl suppository use in the general population are ileus, intestinal obstruction, acute abdominal conditions, including appendicitis, acute inflammatory bowel diseases, severe abdominal pain associated with nausea and vomiting, severe dehydration, and anal fissures or ulcerative proctitis with mucosal damage [50]. Two studies in SCI found that the insertion of rectal medications significantly increased systolic blood pressure [51,52]. This agrees with a retrospective chart review that indicated that rectal medication users had a four-fold increase in the likelihood of reporting autonomic dysreflexia than individuals with SCI, who spontaneously defecated [44]. Care may be necessary when using rectal medications on individuals who are susceptible to autonomic dysreflexia.

An alternative to a suppository, a mini-enema may be used as a first-line rectal medication given that their smaller size and dose may be less irritating and easier to insert. A small tube is inserted, and the liquid contents are squeezed into the rectum. The use of a suppository or mini-enemas may be dependent on local medical practices and reimbursement coverage.

If bowel care is taking too long or is ineffective, then the patient may progress to an enema if the patient is able to self-administer or if a caregiver can assist with administration. Alternatively, a suppository in a water-soluble base (polyethylene glycol) could be considered if that were not already being used. Such PGB suppositories (e.g., Magic Bullet) are generally more expensive but can reduce the time to bowel evacuation by allowing the medication to disperse within minutes after insertion. If bowel evacuation is still taking longer than desired, then one may need to adjust other parts of the bowel program (fluids, fiber, positioning, oral laxatives, etc.).

### 3.8. Narcotics Antagonist

More than 50% of individuals after SCI [53] and MS [54] have chronic pain stemming from neuropathic or musculoskeletal pain. Opioids are still a common choice option for pain management in SCI and MS, especially in refractory cases, although it is increasingly discouraged for non-malignant pain due to its risk for addiction. Opioids, together with immobility, compounds the risk of constipation. No literature was found specific to SCI and opioid-induced constipation or narcotic antagonist. The American Gastroenterological Association (AGA) Guidelines on the Medical Management of Opioid-Induced Constipation [55] recommend laxatives as the first-line agent. In patients with laxative refractory opioid-induced constipation, the AGA recommends using peripherally acting opioid receptor antagonists, which do not enter the central nervous system but block the opioid receptors in the gut (e.g., naloxegol, methylnaltrexone, naldemedine).

## 4. Discussion

The first observation from this study was the stark discrepancy between the large number of agents currently prescribed (Table 1) and an extremely limited amount of literature. Despite the common prescription of oral laxatives and narcotic antagonists, there were no studies with NBD and the best evidence was extracted from idiopathic constipation guidelines, which have serious limitations. There was evidence (low-quality) that polyethylene glycol-based bisacodyl suppositories produced faster outcomes than vegetable-based bisacodyl suppositories. While there was a small amount of literature in SCI, there was little to no literature available for MS. There are few randomized controlled trials evaluating medications for NBD in SCI. Many medications commonly used for NBD are generic and are unlikely to receive large funding for adequate research trials to take place. Given that many of these medications are considered “gold standard”, it is unlikely that there will ever be a study on these medications to compare with placebo given the ethics of withholding gold standard for the sake of research. Only 42% (12/28) of included studies had any control conditions at all (including case–control studies using retrospective data as controls from chart reviews). Thus, it is difficult to make firm assertions based on the research evidence alone, and any results, positive or negative, should be interpreted with caution, taking into consideration any methodological concerns of the study itself.

There are inconsistencies with how NBD is scored between studies. For example, some studies use validated scales, but many rely on self-report (patient bowel journals) to determine bowel dysfunction. Bowel dysfunction in MS is often scored using the Rome criteria [56], but none of the studies we found testing medications on bowel dysfunction used this scale. Standardized and validated measures, such as the International SCI Bowel Function Basic Data Set or the NBD score, used consistently across researchers and clinicians, would produce more detailed descriptions and objective outcomes for comparison [57]. Variations in measurement approaches may be necessary for dysfunction-specific reasons or to meet experimental standards of any particular study, but a key set of bowel measures with a low data collection burden could be used, thus helping researchers and clinicians to embrace collection and reporting of such outcomes [58].

The time period during which bowel dysfunction is measured also varies greatly. We found studies asking participants about their bowel dysfunction over the last week, the last month, the last three months, the last year, or with no interval at all (i.e., have you ever had bowel dysfunction?) Without any decision on what is an appropriate time period to study, we are left with no standard interval for comparison between studies.

### 4.1. Clinical Insights

Because the literature provides little guidance on how and when to prescribe medication for the management of NBD in MS or SCI, we will be providing clinical insight in this section based on our clinical experience and understanding of the literature and guidelines. It is important to remember that pharmacologic treatment is only part of a bowel program for NBD in MS or SCI. As noted in the other manuscripts in this special edition and highlighted in recently published clinical practice guidelines, [22,23] modifications to optimize bowel regulation should not be solely focused on medication changes.

### 4.2. Case 1

History: A 55-year-old female with MS has a power wheelchair and is dependent on transfers and toileting. She has infrequent defecation about 3–5 times per week and abdominal discomfort/bloating. When she has bowel movements, she is able to sense the need to defecate, but she is not able to control the BM (incontinence), and she cannot get to a toilet; thus, the BM occurs in her briefs. She lives with a 65-year-old husband, who is unable to help care for her due to his own health problems. Thus, she has homecare assistance three times per day. When she has a BM into her briefs, she must wait until homecare comes next to get cleaned up. On examination, she has irritation/erythema of the skin of the buttocks with some breakdown and some soiling with stool in the briefs she is wearing. She requires a mechanical lift for transfers and has the weakness of upper limbs, no functional movement in lower limbs, and she needs partial assistance to turn in bed for the exam. She cannot assist at all in lowering pants for examination. She has a relatively preserved sensation of the perineal area and weak anal contraction. There is hard stool present on the rectal exam. She also has significant spasticity in the lower limbs.

Proposed treatment: The main issue here is lack of mobility and independence, thus not being able to toilet when a bowel movement is about to occur. Defecation occurs at times when no assistance is available, leading to being left for up to several hours in soiled briefs with resulting skin breakdown. The second issue is that the infrequency of bowel movements is causing hard stools and discomfort, which may be triggering her spasticity. The goals of treatment would be to have regular, predictable bowel movements, either daily or every second day, in a timely fashion, assisted by her home care workers. If starting with an every-other-day routine, give oral laxative (such as polyethylene glycol 17 mg) every 2 nights, then the next morning administer a rectal bisacodyl suppository, with digital stimulation as needed until the bowel routine is finished. This will allow for a regularly scheduled routine so that bowel incontinence does not occur later when no supports are available and will allow for less discomfort with bloating from infrequent bowel movements. If this approach is not successful, then she may switch the laxative to a more stimulating product, such as sennosides and may switch to a daily schedule if she still has unplanned bowel movements on off days.

### 4.3. Case 2

History: A 35-year-old male who had a traumatic SCI 15 years ago has a C7 AIS A injury. Since the injury, bowel care has consisted of digital anorectal stimulation performed every other day by a caregiver. However, for the last couple of years, the time for bowel care has increased to more than one hour. The patient has episodes of fecal incontinence approximately two times per month. He has vague abdominal discomfort and bloating that makes breathing difficult. Stools are usually hard (type 2 on the Bristol stool chart). For the last year, the patient has taken opioid analgesics because of neuropathic pain and abdominal discomfort.

Proposed treatment: In order to target difficult rectal evacuation and frequent fecal incontinence, first-line treatment will be a stimulant rectal laxative, either as suppository or enema.

In the present case, oral laxatives will most likely be added to counteract symptoms of prolonged colonic transit. The first choice would be an osmotic laxative. If this failed, we would suggest adding a stimulant laxative and, finally, a prokinetic agent.

If there is insufficient relief of symptoms, an opioid antagonist should be prescribed to treat opioid-induced bowel dysfunction. Long-term, additional focus should be given to optimizing this patient’s analgesic regimen using non-opioid options. If the pharmacological treatment failed, consider transanal irrigation or a stoma.

Comments: The case illustrates that NBD usually includes symptoms of constipation as well as fecal incontinence. Treatment with rectal laxatives or an enema is the rational choice as it targets both poor evacuation and fecal incontinence. Patients with spinal cord lesions above the sacral spinal cord often have prolonged transit throughout the colon, which makes oral laxatives or prokinetics a necessary supplement to rectal laxatives. The case also illustrates that NBD is not a stable condition as constipation tends to become increasingly severe with time since injury. Prokinetics and opioid antagonists are usually not prescribed until standard osmotic and stimulant laxatives have failed to provide symptom relief.

### 4.4. Case 3

History: A 65-year-old female had a ground-level fall two years ago that resulted in an injury to the cauda equina. She has bowel movements once or twice per day. Defecation is difficult and usually lasts at least 45 min. Afterward, she has a strong feeling that rectal evacuation was incomplete. Stool consistency is normal. She has no bloating or abdominal pain. Her daily activities are restricted by the need to keep near a toilet because she has fecal incontinence several times per week. She has no other significant medical problems. On examination, there is reduced perianal sensation and very weak voluntary contraction of the anal canal.

Proposed treatment: The first choice of treatment would be a stimulant rectal laxative administered daily, preferably in the morning, to keep her continent during the day. If this failed, the patient should be offered transanal irrigation.

Comments: Lesions at the conus medullaris or cauda equina often cause poor evacuation of the rectum as well as fecal incontinence. In most cases, transport through the proximal colon is less severely affected. Rational treatment aims at restoring rectal evacuation by rectal laxatives (suppositories or enema) or by transanal irrigation. Oral laxatives are usually not needed unless stools are hard, and then they would be prescribed.

### 4.5. Recommendations for Future Research

Researchers have suggested that to increase the data quality and effectiveness of clinical research studies, the use of large data sets (like SCI model systems) can facilitate comparisons among treatments, patients, centers, and countries [59]. As SCI and MS are technically “lower frequency” conditions compared to stroke, cancer, or heart disease, it can be difficult to get sample sizes that are large enough to have any statistical power. The SCI model systems database network has helped contribute to research with greater statistical power, and thus we can have more confidence in results that are generalizable.

Some additional suggestions for areas in which SCI and MS research can improve include:

- Matched control research would increase the number of studies with a control group and would also help to establish sorely needed norms in SCI and MS research. Both neurological diseases affect many-body systems and understanding what norms are for individuals with NBD for colon transit time, bowel evacuation time and frequency after nutritional additions, an exercise intervention, or medication changes would be extremely useful;
- Standardizing a bowel treatment training program and evaluating learning and behavioral changes. Education research is rare, and the components of what constitutes a quality bowel training program have not yet appeared in the published literature;
- Research on the long-term effects of bowel medications or medications to reduce side-effects in NBD is much needed. Individuals with NBD can experience more severe bowel-related symptoms over time, although it is not known whether this is due to aging, medications becoming less effective, or the development of conditions, such as megacolon (colonic dilatation) [60];
- Research on biomarkers that precede constipation, incontinence, or more serious bowel problems, such as fecal impaction.

## Figures and Tables

**Table 1:** Current Medications used in neurogenic bowel dysfunction (NBD), including mechanism of action.

Generic Names	Examples of Trademark Names	Mechanism of Action
Oral Laxatives		
Polyethylene glycol (PEG)	Miralax, Movicol, Restorolax, Lax a Day	Osmotic laxative
Magnesium hydroxide	Milk of Magnesia	Osmotic laxative
Docusate sodium	Colace, Surfak	Osmotic laxative
Lactulose	Lactulose, Kristalose	Osmotic laxative
Bisacodyl	Dulcolax	Stimulant laxative
Sennosides	ExLax, Senokot	Stimulant laxative
Rectal Laxatives		
Polyethylene glycol (peg)	Glycolax (suppository)	Osmotic laxative
Sodium citrate	Microlax (micro enema; also includes sodium lauryl and sorbitol)	Osmotic laxative
Bisacodyl	Dulcolax (suppository), Magic Bullet (suppository)	Stimulant laxative
Sennosides	Senokot (suppository)	Stimulant laxative
Docusate sodium	Colace (glycerin suppository or micro enema), Surfak, Enemeez (mini enema)	Stool softener laxative
Prokinetic drugs		
Prucalopride	Resotran, Resolor	Oral serotonin HT4 agonist with prokinetic properties
Secretory		
Linaclotide	Linzess or Constella	Oral guanylate cyclase-c agonist, which increases intestinal secretions
Narcotic Antagonists		
Naloxegol	Movantik, Movantig	Oral opioid antagonist
Lubiprostone	Amitiza	Oral opioid antagonist
Methylnaltrexone bromide	Relistor	Oral or subcutaneous injection opioid antagonist

## Supplementary Materials

The following are available online at https://www.mdpi.com/2077-0383/10/4/882/s1, S1. Search Terms, S2. Results of the Literature Search and S3. Results by Medication, Quality Ratings and Risk of Bias Assessment.
