# Supplementary material for: Pharmacological Management of Neurogenic Bowel Dysfunction after Spinal Cord Injury and Multiple Sclerosis: A Systematic Review and Clinical Implications
Source: J Clin Med. 2021 Feb 22;10(4):882. doi: 10.3390/jcm10040882 (PMC7926827; doi:10.3390/jcm10040882)
Supplement: Supplementary file 1 [file jcm-10-00882-s001.pdf]

Supplementary Materials

S1. Search terms

Extraction Note: no studies were found pertaining to the remaining medications in the SCI population. This included: oral Bisacodyl, Naloxegol, Lubiprostone, magnesium Hydroxide, Docusate Sodium, Sennosides, Lactulose, Linaclotide, and Methylnaltrexone Bromide (prucalopride OR Resolor OR Resotran) AND (Spinal cord injur or quadripleg* or parapleg* or spinal cord impaired or spinal cord lesion or neurogenic bowel) (prucalopride OR Resolor OR Resotran) AND (multiple sclerosis)

(Bisacodyl OR Dulcolax OR Carters Little Pills) AND (Spinal cord injur* or quadripleg* or parapleg* or spinal cord impaired or spinal cord lesion or neurogenic bowel) (Bisacodyl OR Dulcolax OR Carters Little Pills) AND (multiple sclerosis) AND (Bowel or constipation or defecation or defecate or neurogenic bowel or constipation or incontinence) (Polyethylene glycol OR RestoraLAX OR Lax-A—Day OR Pegalax OR Miralax OR macrogol OR movicol OR softlax OR clearLAX OR osmolax OR glycolax OR restoraLAX OR Lax-a-Day OR pegalax OR PEG) AND (Spinal cord injur* or quadripleg* or parapleg* or spinal cord impaired or spinal cord lesion or neurogenic bowel)(Polyethylene glycol OR RestoraLAX OR Lax-A Day OR Pegalax OR Miralax OR macrogol OR movicol OR softlax OR clearLAX OR osmolax OR glycolax OR restoraLAX OR Lax-a-Day OR pegalax OR PEG) AND (multiple sclerosis) AND (Bowel or constipation or defecation or defecate or neurogenic bowel or constipation or incontinence) (movantic or moventig or naloxegol) AND (Spinal cord injur* or quadripleg* or parapleg* or spinal cord impaired or spinal cord lesion or neurogenic bowel) (movantic or moventig or naloxegol) AND (multiple sclerosis) (Amitiza or lubiprostone) AND (Spinal cord injur* or quadripleg* or parapleg* or spinal cord impaired or spinal cord lesion or neurogenic bowel)

(Amitza or lubiprostone) AND (multiple sclerosis)

(Milk of Magnesia or Magnesia or magnesium Hydroxide or magnesium) AND (Spinal cord injur* or tetrapleg* or quadripleg* or parapleg* or spinal cord impaired or spinal cord lesion) AND (Bowel or constipation or defecation or defeacate or neurogenic bowel or constipation or incontinence) (Milk of Magnesia or Magnesia or magnesium Hydroxide or magnesium) AND (multiple sclerosis) AND (Bowel or constipation or defecation (Enemeez OR docusate sodium OR Enemeez Plus OR Therevac mini enemas OR Therevac-SB OR docusate OR Colace OR Diocto Dioeze OR Doc-Q-Lace OR Docu OR Docu Soft OR Doculase OR Docuprene OR Docusil OR Docusoft S OR DocuSol OR DOK OR DSS OR Dulcolax Stool Softener OR Enemeez Mini OR Kao-Tin OR Octycine-250 OR Pedia-Lax Stool Softener OR Phillips Stool Softener OR Promolaxin OR Silace OR Surfak Stool Softener OR Sur-Q-Lax OR Vacuant OR dioctyl sulfosuccinate) AND (Spinal cord injur* or quadripleg* or parapleg* or spinal cord impaired or spinal cord lesion or neurogenic bowel)

(Enemeez OR docusate sodium OR Enemeez Plus OR Therevac mini enemas OR Therevac-SB OR docusate OR Colace OR Diocto Dioeze OR Doc-Q-Lace OR Docu OR Docu Soft OR Doculase OR Docuprene OR Docusil OR Docusoft S OR DocuSol OR DOK OR DSS OR Dulcolax Stool Softener OR Enemeez Mini OR Kao-Tin OR Octycine-250 OR Pedia-Lax Stool Softener OR Phillips Stool Softener OR Promolaxin OR Silace OR Surfak Stool Softener OR Sur-Q-Lax OR Vacuant OR dioctyl sulfosuccinate) AND (multiple sclerosis) AND (Bowel or constipation or defecation or defecate or neurogenic bowel or constipation or incontinence) (Senna or Ex-lax or Senokot or SenoSol) AND (Spinal cord injur* or quadripleg* or parapleg* or spinal cord impaired or spinal cord lesion or neurogenic bowel)(Senna or Ex-lax or Senokot or SenoSol) AND (multiple sclerosis)(lactulose) AND (Spinal cord injur* or quadripleg* or parapleg* or spinal cord impaired or spinal cord lesion or neurogenic bowel)

(lactulose) AND (multiple sclerosis)

(Linzess or linaclotide) AND (Spinal cord injur* or quadripleg* or parapleg* or spinal cord impaired or spinal cord lesion or neurogenic bowel)

(Linzess or linaclotide) AND (multiple sclerosis)

(Relistor or methylnaltrexone) AND (Spinal cord injur* or tetrapleg* or quadripleg* or parapleg* or spinal cord impaired or spinal cord lesion or neurogenic bowel or neurogenic bowel dysfunction) (Relistor or methylnaltrexone) AND (multiple sclerosis) (Glycerin OR Colace Glycerin Suppositories OR Fleet Babylax OR Fleet Glycerin Suppositories Adult OR

Fleet Glycerin Suppositories Pediatric OR Glycerin Suppositories Maximum Strength OR Pedia-Lax Liquid OR Sani-Supp) AND (Spinal cord injur* or tetrapleg* or quadripleg* or parapleg* or spinal cord impaired or spinal cord lesion or neurogenic bowel or neurogenic bowel dysfunction)(Glycerin OR Colace Glycerin Suppositories OR Fleet Babylax OR Fleet Glycerin Suppositories Adult OR Fleet Glycerin Suppositories Pediatric OR Glycerin Suppositories Maximum Strength OR Pedia-Lax Liquid OR Sani-Supp) AND (multiple sclerosis) (Phosphate enema OR Sodium phosphate OR Fleet enema) AND (Spinal cord injur* or tetrapleg* or quadripleg* or parapleg* or spinal cord impaired or spinal cord lesion or neurogenic bowel)(Phosphate enema OR Sodium phosphate OR Fleet enema) AND (multiple sclerosis) AND (Bowel or constipation or defecation or defecate or neurogenic bowel or constipation or incontinence)

S2. Results of Literature searching

PRISMA Flow Diagram

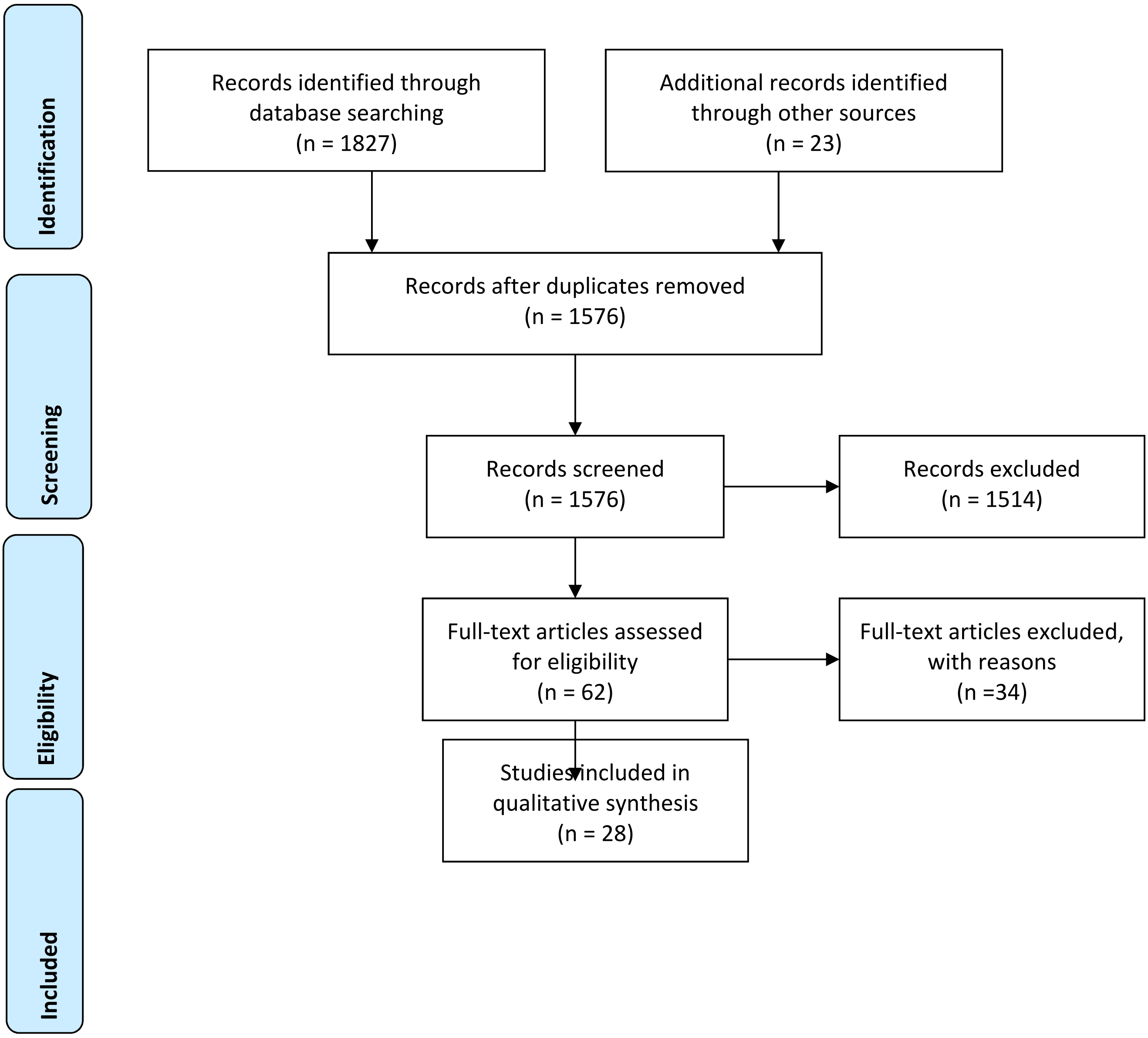

S3. Results by Medication, Quality Ratings and Risk of bias assessment.

Table 1a. Data abstraction of prucalopride in SCI.

Author, Year Study Design Setting	Population Characteristics Sample Size		Interventions	Outcomes	Results
	SCI	Control			
**Krogh 2002**<br><br>**RCT (Double-blinded, Phase II)**<br><br>**University Hospital Arhus, Denmark**	**Prucalopride 1 mg**<br>**N: 8**<br>**Level: excluded cauda equina lesions**<br>**Age:** mean 36.5 y, SD 3.91 y<br>**Duration:** mean 13.6 y, SD 2.15 y<br>**% Female:** 37.5%<br><br>**Prucalopride 2 mg**<br>**N: 8** (4 withdrew; some data still included from these participants)<br>**Level:** excluded cauda equina lesions<br>**Age:** mean 44.3 y, SD 3.05 y<br>**Duration:** mean 11.6 y, SD 3.08 y<br>**% Female:** 28.6	**Intervention 1&2 (placebo):**<br>**N:** 7 (1 withdrew)<br>**Age:** mean 34.7 y, SD 2.49<br>**Duration:** 14.3 y, SD 2.75 y<br>**% Female:** 14.3%	**Intervention 1:** daily prucalopride 1 mg or placebo for 4 w<br>**Intervention 2:** daily prucalopride 2 mg or placebo for 4 w<br><br>**Comparing:** before (4 w run-in period) vs. after, placebo vs. prucalopride 1 mg, and prucalopride 2 mg vs. placebo<br><br>**Prucalopride complications:** flatulence (4 patients in 1 mg group), bradycardia (2 patient in 1 mg group vs. 4 in 2 mg group), headache (1 patient in 1 mg group vs. 4 in 2 mg group), abdominal pain (3 patients in 2 mg group), skin disorder (2 patients in placebo vs. 1 in 2 mg group), constipation (1 patient in placebo vs. 1 in 1 mg group), diarrhea (1 patient in 1 mg group vs. 2 in 2 mg group), hyperphosphatemia (1 patient in placebo vs. 1 in 2 mg group)<br><br>**Reasons for withdrawals (Total 5):** abdominal pain and headache (2 patients, 2 mg group), pressure sore in gluteal region (1 patient, 2 mg group; considered unrelated to treatment), diarrhea and headache (1 patient, 2 mg group), fecal incontinence (1 patient, placebo)	**Timeline:** 1997–1998<br><br>**Source:** X-ray analysis, diary entries, visual analog scales, lab values<br><br>**Outcomes:** time of toilet trip; use of anal stimulation; consistency; use of digital evacuation; time taken for defecation; presence of fecal impaction; date and time of medication intake; any rescue laxative usage; presence and intensity of side effects abdominal pain/cramps, abdominal bloating/distension, and presence of help needed from carer. Constipation severity, ECG and clinical laboratory parameters (hematology, biochemistry, and urinalysis), heart rate, blood pressure and adverse events	1. Self-reported number of bowel movements changed on weekly average by -0.6 (95% CI -2.5 to 0.8) after placebo, 0.0 (95% CI -1.8 to 1.0) after prucalopride 1 mg group, and 0.6 (95% CI 0.2 to 1.2) after prucalopride 2 mg group.<br>2. Constipation severity decreased after prucalopride treatment. The VAS score for treatment efficacy showed a clear dose-response (medians 4, 52, and 73 for placebo, 1 and 2 mg, respectively).<br>3. Median decrease by 38.5 h in CTT after 4 w of treatment with prucalopride 2 mg compared with run-in (95% CI -80 to -5

Table 1b. Grading of body of evidence—prucalopride in SCI.

# Studies (design)	Risk of Bias	Inconsistency	Indirectness	Imprecision	Point Estimate(s)	Quality of Evidence Rating (GRADE)
1 (RCT)	Very serious<br>*unclear group assignment<br>*missing patient data	Very serious<br>*Single RCT, so large confidence interval	Not serious	Serious<br>*Less than 400 participants	1 mg prucalopride:<br>1. Weekly bowel movement increase of 0.0 (95% CI -1.8 to 1.0)<br>2. Subjective treatment efficacy score 52/100<br><br>2 mg prucalopride:<br>1. Weekly bowel movement increase of 0.6 (95% CI 0.2 to 1.2)<br>2. Subjective treatment efficacy score 73/100<br>3. Median decrease by 38.5 h in CTT (95% CI -80 to -5 h)	Very low

Table 1c. Risk of bias for randomized control trials of prucalopride in SCI.

Author, Year	Randomization adequate?	Allocation concealment adequate?	Groups similar at baseline?	Eligibility criteria specified?	Participants and personnel blinded?	Outcome assessors blinded?	No evidence of unreported outcomes?	Attrition rate below 15%?	Analyzed according to randomization?	Risk of Bias
Krogh, 2002	Unclear	Unclear	Yes	Yes	Yes	Yes	No<br>*patient and outcome data missing	No<br>*50% of the 2 mg group withdrew	No<br>*missing data	High

Table 2a. Data abstraction—metoclopramide in SCI.

Author, Year Study Design Setting	Population Characteristics		Interventions	Outcomes	Results
	SCI	Control			
**Segal, 1987**<br><br>**Prospective Controlled Trial**<br><br>**VA Medical Center, California, USA**	**N:** 20 (11 respondents)<br>**Level:** 11 tetraplegics, 9 paraplegics; all complete<br>**Etiology:** traumatic<br>**Age:** range 20–55 y<br>**Duration:** >1 y<br>**% Female:** 0%	Controls defined as spinally intact people<br>**N: 8**	**Intervention:** 2–3 mins intravenous infusion of metoclopramide (10 mg)<br><br>**Comparing:** before vs. after and SCI vs. spinally intact individuals	**Source:** time-course profile and rate of the disappearance of isotopically labeled liquid meal, anterior scintigraphy<br><br>**Outcomes:** half time of gastric emptying ($GE_{1/2}$) and gastric emptying patterns in the early and later phases (overnight fasting; water syrup mixture combined with medical radioisotopes was ingested; a gamma camera used for imaging for 120 minutes)	1. After metoclopramide, mean $GE_{1/2}$, for a liquid meal decreased in tetraplegics (from 104.8 to 18.8 min; $p < 0.004$) and paraplegics (from 111.5 to 29.1 min; $p < 0.04$).<br>2. Positive association between reduction in $GE_{1/2}$ and delay in gastric emptying before treatment ($p < 0.05$).<br>3. Inverse association ($r > -0.5$, $p < 0.05$) between duration of injury and $GE_{1/2}$.<br>4. Positive correlation between $GE_{1/2}$ and level of SCI ($p < 0.05$).
Miller and Fenzl, 1981<br><br>Case Study	**N:** 1<br>**Level:** c-5 complete quadriplegic<br>**Etiology:** traumatic<br>**Age:** 21 y<br>**Duration:** 12 w<br>**% Female:** 0%	None	**Intervention:** 2 w of 10 mg intravenous metoclopramide twice daily, 3 w of 10 mg oral metoclopramide prior to each meal and 2 w of 5 mg oral metoclopramide prior to each meal	**Source:** clinical evaluation<br><br>**Outcome:** resolution of ileus	1. Ileus was resolved

Table 2b. Grading of body of evidence—metoclopramide.

# Studies (design)	Risk of Bias	Inconsistency	Indirectness	Imprecision	Point Estimate(s)	Quality of Evidence Rating (GRADE)
2 (Obs)	Very serious *no controls and case study	Serious *Outcomes do not match	Serious *gastric emptying may not be correlated to bowel care	Serious *less than 400 participants	Reduces gastric emptying from 106.6 to 21.6 minutes (p < 0.006)<br><br>Resolves ileus (n=1)	Very low

Table 2c. Risk of bias—Observational studies—metoclopramide in SCI.

Author, Year	Report clear review question, state inclusion and exclusion of primary studies?	Substantial effort to find relevant research?	Adequate assessment of validity of included studies?	Sufficient detail of individual studies presented?	Primary studies summarized appropriately?	Risk of Bias
Segal, 1987	No *Only report on responders	Yes	Yes	Yes	Yes	No
Miller and Fenzl, 1981	No *single patient	No	No	No	Unclear	No

Table 3a. Data abstraction—neostigmine.

**Author, Year Study Design Setting**	**Population Characteristics**		**Interventions**	**Outcomes**	**Results**
	**SCI**	**Control**			
Rosman, 2008<br><br>RCT (cross over)<br><br>USA	**N:** 7<br>**Level:** 4 cervical, 3 thoracic<br>**Age:** mean 46.9 y, range 30–56 y<br>**Duration:** mean 15.9 y, range 3–27 y<br>**Ethnicity:** 2 Hispanic, 5 African American	Placebo Control Same population	**Interventions:** either intramuscular injections of 2 mg neostigmine and 0.4 mg glycopyrrolate or placebo during 3 consecutive bowel evacuation sessions. Crossover after a 1 w washout period in between each intervention.<br><br>**Comparing:** neostigmine and glycopyrrolate vs. placebo<br><br>**Neostigmine and glycopyrrolate complications:** 57% experienced dry mouth, 57% experienced twitching, 14% experienced excess salivation, and 29% experienced abdominal cramps (compared to 0%, 29%, 0%, and 43% in placebo, respectively. No side effects persisted longer than 60 mins.	**Source:** physical evaluations and questionnaire<br><br>**Outcomes:** total bowel evacuation time, time to first flatus, time to beginning of stool flow, time to end of stool flow, Pulse, blood pressure, heart rate, and side effects	1. Compared with placebo, neostigmine/glycopyrrolate reduced total bowel evacuation time from 98.1 to 74.8 ($p < 0.05$).<br>2. Neostigmine/ glycopyrrolate reduced time to first flatus (56.9 min to 21.8 mins; $p = 0.001$), time to beginning of stool flow (69.8 min to 42.3 min; $p = 0.01$) and time to end of stool flow (80.3 to 53.3 min; $p < 0.05$).<br>3. Neostigmine/ glycopyrrolate resulted in slightly decreased lowest heart rate (67.6 vs. 64.6 beats/min, $p = 0.4$), but no significant difference in blood pressure.
Korsten, 2005<br><br>RCT (cross over)	**N:** 13<br>**Level:** C4-T12; 12/13 motor complete, 5/13 sensory incomplete; 5	Control defined as saline intravenous infusions.	**Interventions:** intravenous 2 mg neostigmine or 2 mg neostigmine + 0.4 mg glycopyrrolate<br><br>**Comparing:** sessions with 2 mg neostigmine vs. 2 mg neostigmine + 0.4 mg glycopyrrolate vs. normal saline	**Source:** clinical examination<br><br>**Outcomes:** bowel evacuation (barium paste), blood pressure, pulse,	1. Both interventions had a greater expulsion of stool than with saline ($p < 0.01$).<br>2. Mean time of expulsion was 11.5 min after neostigmine and 13.5 min

New York, USA	tetraplegics, 8 paraplegics<br>**Age:** mean 46 y, range 25–69 y<br>**Duration:** mean 14 y, range 1–31 y	Same population	**Neostigmine Complications:** total airway resistance (27%), central airway resistance (17%), facial and tongue fasciculations (92%)<br><br>**Neostigmine and glycopyrrolate complications:** facial and tongue fasciculations (89%)<br><br>**Both group complications:** diaphoresis, salivation, and abdominal cramping, which occurred in subjects with injuries below thoracic level 10. Side effects persisted less than 30 min post-infusion.	total and central airway resistance, and side effects	after neostigmine and glycopyrrolate.<br>3. No correlation between level of SCI and likelihood of bowel evacuation.
Korsten, 2018<br><br>Phase I Clinical Trial<br><br>James J. Peters Veterans Affairs Medical Center, NYC USA	**N:** 25<br>**Level:** 15 paraplegic, 10 tetraplegic; 11 cervical, 13 thoracic, and 1 lumbar; ASI Score: 12 A, 3 B, 7 C, 3 D<br>**Age:** mean 50 y, SD 15 y<br>**Duration:** mean 9 y, SD 9 y	None	**Interventions:** individuals were screened for responsiveness to intravenous neostigmine (0.03 mg/kg)/glycopyrrolate (0.006 mg/kg). Intravenous responders were administered low-dose transdermal neostigmine (0.05 mg/kg) /glycopyrrolate (0.01 mg/kg) by iontophoresis. Nonresponders to the low dose were administered high-dose transdermal neostigmine (0.07 mg/kg) /glycopyrrolate (0.014 mg/kg) by iontophoresis. Maxed out at 10.0 mg neostigmine and 2.0 glycopyrrolate.<br><br>**Comparing:** intravenous neostigmine (IV) vs. low dose transdermal (low TD) vs. high dose transdermal (high TD)	**Source:** clinical evaluation<br><br>**Outcomes:** vital signs (blood pressure, heart rate, and pulse oximetry), bowel movement, bowel evacuation time, and cholinergic side effects	1. 21/25 responded to IV; 5 of the 21 IV responders had a bowel movement with low TD; 5 of the 16 of low TD nonresponders had a bowel movement with high TD. 11 of 21 IV responders did not respond to either low TD or high TD.<br>2. No significant difference in bowel evacuation time among the three doses (IV: 21, low TD: 36 mins, high TD: 26).<br>3. The response rate to high and low TD combined was lower than

					IV but not significant (40% vs. 75%).<br>4.

Table 3b. Grading of body of evidence—neostigmine.

# Studies (design)	Risk of Bias	Inconsistency	Indirectness	Imprecision	Point Estimate(s)	Quality of Evidence Rating (GRADE)
2 RCT	Serious<br>*does not disclose any information about randomization process.	Not serious<br>*cannot pool data, large confidence interval	Not serious	Serious<br>*Less than 400 participants	N+G<br>1. Initiated evacuation after 42.3 minutes<br>2. Evacuation times of 11–21 minutes<br>3. Increased expulsion of stool	Very low
1 (Obs)	Very serious	Very serious<br>*different outcome measures. Cannot pool data, large confidence interval	Not serious	Serious<br>*less than 400 participants		Very low

Table 3c1. Risk of bias, randomized control trials—neostigmine.

Author, Year	Randomization adequate?	Allocation concealment adequate?	Groups similar at baseline?	Eligibility criteria specified?	Participants and personnel blinded?	Outcome assessors blinded?	No evidence of unreported outcomes?	Attrition rate below 15%?	Analyzed according to randomization?	Risk of Bias
Rosman, 2008	Unclear	Yes	Yes	Yes	Unclear	Unclear	Unclear	Yes	Unclear	Moderate
Korsten, 2005	Unclear	Unclear	Yes	Yes	Yes	Yes	Unclear	Yes	Yes	Moderate

Table 3c2. Risk of bias, observational studies—neostigmine.

Author, Year	Non-biased selection?	Attrition rate below 15%?	Outcomes prespecified and defined?	Methodology adequately described?	Non-biased and adequate methodology?	Statistical analysis of potential confounds?	Adequate duration of follow-up?	Risk of Bias
Korsten, 2018	No *Only report on responders	Yes	Yes	Yes	Yes	No	N/A	High *bias selection

Table 4a. Data abstraction of suppositories and enemas.

Author, Year<br>Study Design<br>Setting	Population Characteristics		Interventions	Outcomes	Results
	SCI	Control			
Hwang, 2017<br><br>Longitudinal Cohort<br><br>Shriners Hospitals for Children, USA	**Database** N: 464 (131 examined)<br>**Level:** 77 tetraplegic, 54 paraplegic; 100 complete, 31 incomplete<br>**Etiology:** 118 traumatic (67 vehicular/pedestrian, 7 violence, 11 falls/flying objects, 33 sports), 12 medical/surgical, and 1 unknown/other<br>**Ethnicity:** 112 Caucasian, 5 African American, 1 American Indian, 11 Hispanic, and 2 Asian<br>**Age:** mean 33.4 y, SD 6.1 y<br>**Duration:** mean 19.5 y, SD 7.0 y (all pediatric-onset)<br>**% Female:** 35.9%	None	**Objective:** describe long-term NBD management programs, including changes, and assess psychosocial impact	**Timeline:** Jan 2010–Sep 2015<br>Follow-up after:<br>**Range:** 3–5 y<br><br>**Source:** at least 2 annual telephone interviews, questionnaires and medical records<br>**Outcomes:** type and time of bowel program, bowel and abdominal symptoms, Craig Handicap Assessment and Reporting Technique (CHART), satisfaction with life scale, patient health questionnaire-9, and Short Form-12	1. Rectal suppositories/enemas were the most common bowel program (44.3% at last follow-up)<br>2. Based on the reports, likelihood of a patient using rectal suppositories/ enemas decreased by 6.7% each y ($p = 0.01$; OR 0.933, 95% CI 0.896–0.973).<br>3. From reports, paraplegics were 90% less likely to continue using rectal suppositories/enema compared to tetraplegics ($p < 0.001$; OR 0.099 CI 0.046–0.213).

Furusawa, 2009<br><br>Prospective controlled trial<br><br><br>Kibikogen Rehabilitation Center for Employment Injuries, Japan	**N:** 25 (Not NBD specific)<br>**Level:** cervical (5 C4, 8 C5, 7 C6, 5 C7); AIS Score: 20 A 5 B<br>**Age:** mean 32.3 y, range 18–58 y<br>**Duration:** mean 23.4 mo, range 3–172 mo<br>**% Female:** 12%	Control defined as placebo jelly applied to anal canal.	**Intervention:** manual evacuation and DRS with 10 mL of placebo or 2% lidocaine applied to the anal canal<br><br>**Comparing:** before vs. during vs. after, and intervention vs. control	**Timeline:**<br>Follow-up after: 5 minutes, 30 minutes and 24 h<br><br>**Source:** it was conducted in a clinical setting with an automated vital sign-recorder placed around right arm while patient was in a recumbent position. A nurse stopped the intervention if BP reached 160 mmHg.<br>**Outcomes:** BP, H, and AD symptomology	Placebo group:<br>1. Increase in sBP when rectal meds were inserted ($p < 0.01$), which remained elevated until 5 minutes defecation.<br>2. Had a higher diastolic and sBP during DRS than lidocaine group (both $p < 0.01$)<br>3. Had a higher maximal increase in sBP than the lidocaine group (mean 50.2 vs. 33.2 mmHg; $p < 0.001$).<br><br>**AD Symptoms (10 patients)**:<br>Control group: 6 headache, 5 flushings, and 1 goosebumps<br>Lidocaine group: 2 headaches, 1 flushing and 1

Furusawa, 2011<br><br>Retrospective Chart Review<br><br>28 Rosia Hospitals, Japan	**N:** 571<br>**Level:** all above or at T6; 181 C1-4, 346 C5-8, 27 T1-T4, and 17 T5-6; AIS Score: 29.4% A 6.1% B 22.2% C 42.2% D<br>**Etiology:** traumatic (242 motor vehicle collision, 240 falls, 32 sports-related injuries, 21 direct, blunt injuries, and 36 other)<br>**% Female:** 18.4%	None	**Objective:** investigate the relationship between the different bowel and bladder management methods and the incidence of AD during hospitalization in SCI patients<br>**Comparing:** AD across age, gender, injury level, and bladder and bowel management techniques	**Timeline:** Apr 1997–Mar 2007<br><br>**Source:** hospital registry database<br>**Outcomes:** AD and bowel and bladder management techniques	Rectal medication users:<br>1. 37.1% of patients were users<br>2. Had an increased likelihood of AD than spontaneous defecation users (OR 4.37, 95% CI 2.30–8.31; $p < 0.001$). Prevalence of symptomatic AD (27.4%) among users was only second-highest to manual evacuation users (39.4%, OR 7.56)<br>3. Reported a longer duration of hospitalization than users of spontaneous defecation (265 vs. 201 days; $p < 0.001$)<br><br>**Complications:** 39.4% had symptomatic AD (elevation of sBP with headache, sweating or flushing above the level of injury, nasal congestion, blurred vision and anxiety.

Furusawa, 2007<br><br>Pre-post<br><br>Japan	**N:** 15 (Not NBD specific)<br>**Level:** all cervical; AIS 13 A, 2 B<br>**Age:** mean 40.9 y, SD 15.4 y<br>**Duration:** mean 45.8 y, SD 42.0 mo<br>**% Female:** 6.7%	Control defined as no bowel progra m done in the identical posture (lateral recumb ency)	**Intervention:** manual evacuation and DRS<br>**Comparing:** before (5 minutes) vs. during vs. after and<br>control vs. intervention	**Timeline:** Follow-up after: 5 minutes, 30 minutes and 24 h<br><br>**Source:** it was conducted in a clinical setting with an automated vital sign-recorder placed around right arm while patient was in a recumbent position. A nurse paused the intervention if sBP reached 160 mmHg.<br>**Outcomes:** BP, pulse rate, and AD symptoms (defined cardiovascular AD as 20–40 mmHg increase in baseline sBP)	1. Insertion of rectal meds induced an increase in sBP, which persisted during additional DRS ($p < 0.01$).<br>2. sBP recovered to baseline values within 5 minutes after defecation.<br>3. Insertion of a finger into the anal canal after the end of stool flow did not increase BP.<br><br>**AD symptoms (5 patients):** chills (2 patients), goosebumps (2 patients), sweating (1 patient), and headache (2 patients)

Inskip, 2018<br><br>Cross-sectional<br><br>Survey of Greater Vancouver Area, Canada and Multinational Digital Survey	**N:** 287 (73% completed all survey items)<br>**Level:** 45% cervical, 45% thoracic, 9% lumbar, 1% sacral; 70% incomplete; 57% at or above T7<br>**Age:** mean 49.2 y, SD 13.2 y<br>**Duration:** mean 17.1 y, SD 12.9 y	None	**Objective:** describe the presence of cardiovascular symptoms during bowel care, impact of bowel management and associated cardiovascular concerns on QoL<br><br>**Comparing:** bowel management techniques used and outcomes	**Timeline:** 3 y **Source:** bowel care and cardiovascular function questionnaire **Outcomes:** bowel care management, cardiovascular symptoms, nutrition, hydration, medication use, and participant demographics	1. Of those at risk for AD (T7 or above; n = 163), 74% had AD symptoms during bowel care, and 32% described palpitations. AD interfered with activities of daily living in 51%.<br>2. Longer durations of bowel care (p < 0.001) and more severe AD (p = 0.04) were associated with lower QoL.<br>3. Those who used enemas and suppositories reported more impaired QoL than those using other methods of bowel management.
Adriaansen, 2015<br><br>Multicenter Cross-sectional<br><br>Netherlands	**N:** 282 (258 completed the questionnaire)<br>**Level:** 40% tetraplegic; AIS Score: 70% A 12% B 9% C 9% D<br>**Etiology:** 90% traumatic<br>**Age:** mean 48 y, range 29–65 y<br>**Duration:** mean 24 y, range 10–47 y<br>**% Female:** 27%	None	**Objective:** describe neurogenic bowel management and its outcomes in individuals living with a SCI for at least 10 y	**Timeline:** Nov 2011–Feb 2014 **Source:** clinical assessments, oral interviews, and questionnaire **Outcomes:** international SCI Bowel Function Basic Data Set, NBD score, and satisfaction with bowel management	1. Participants experiencing severe NBD were more likely to report use of suppositories (p = 0.002, OR= 2.99)<br>2. Participants experiencing >60 minutes for defecation (38.3%) were more likely to report suppository use (p < 0.001, OR= 8.11)<br>3. Suppository use was a predictor of severe NBD (OR 4.02; p < 0.001).

Coggrave, 2009<br><br>Cross-sectional<br><br>National Spinal Injuries Center, UK	**N:** 1334 respondents<br>**Level:** 541 cervical (218 complete, 289 incomplete, 34 unknown), 669 thoracic (399 complete, 207 incomplete, 63 unknown); 112 lumbar (23 complete, 70 incomplete, 19 unknown), 4 sacral (1 incomplete, 3 unknown)<br>**Etiology:** traumatic or acute onset disease<br>**Age:** median 51.5 y, range 19–91 y<br>**Duration:** median 17.6 y, range 1–65 y<br>**% Female:** 26.6%	None	**Objective:** describe bowel management in community-dwelling SCI individuals and to explore associations between age, injury, dependency, problems, interventions, and satisfaction	**Source:** mail-in questionnaire<br><br>**Outcome:** method of evacuation, number of interventions used before finding a successful protocol, bowel care problems and assistance with bowel care	Enema related results:<br>Associated with the following in cervical injuries (C), thoracic injuries (T) and lumbar/sacral (L/S):<br>a. fecal incontinence (p = 0.01)<br>b. rectal prolapse (p = 0.01)<br>c. abdominal pain (p = 0.01, OR 2)<br>d. constipation C (p = 0.003, OR 2.8)<br>e. longer duration (p < 0.001)<br>f. AD (p = 0.002)

Table 4b. Risk of bias for observational studies of suppositories and enemas.

**Author, Year**	**Non-biased selection?**	**Attrition rate below 15%?**	**Outcomes prespecified and defined?**	**Methodology adequately described?**	**Non-biased and adequate methodology?**	**Statistical analysis of potential confounds?**	**Adequate duration of follow-up?**	**Risk of Bias**
Inskip, 2018	Unclear<br>*open access digital survey	Yes	Yes	Yes	Yes	Yes	N/A	High<br>*open access digital survey
Hwang, 2017	Unclear<br>*does not state why only 131 patients were included from 464 patient data set	Unclear	Yes	Yes	Yes	No	Yes	High<br>*unclear selection criteria

Adriaansen, 2015	Yes	Yes	Yes	Yes	Yes	Yes	N/A	Low
Furusawa, 2011	Yes	Yes	Yes	Yes	Yes	No	N/A	Moderate
Coggrave, 2009	No<br>*45.1% response rate	Yes	Yes	Yes	Yes	No	N/A	High<br>*45.1% response rate
Furusawa, 2009	Yes	Yes	Yes	Yes	Yes	No	N/A	Moderate
Furusawa, 2007	Unclear<br>*sampling source not stated	Yes	Yes	Yes	Yes	No	N/A	Moderate

Table 4c. Grading of body of evidence—suppositories and enemas.

# Studies (design)	Risk of Bias	Inconsistency	Indirectness	Imprecision	Point Estimate(s)	Quality of Evidence Rating (GRADE)

7 (Obs)	Very serious *only 2 control groups	Very serious *Broad definition of interventions *unclear if symptoms proceed or come before intervention use	Not serious	Not serious	Individuals who reported Rectal Medication use:<br>1. More likely to report impaired QoL than those who used alternative bowel techniques 2. More likely to be experiencing AD than spontaneous defecation users (OR 4.37, 95% CI 2.30–8.31; p < 0.001)<br><br>Individuals who reported enema use:<br>1. More likely to be experiencing fecal incontinence (p = 0.01)<br>2. And had cervical injuries were more likely to be experiencing constipation (p = 0.003, OR 2.8) 3. More likely to be experiencing longer bowel session (p < 0.001).<br><br>Individuals who reported suppository use:<br>1. More likely to be experiencing severe NBD (p = 0.002, OR= 2.99)<br>2. More likely to be experiencing > 60 minutes for defecation (p < 0.001, OR= 8.11)	Very low

Table 5a1. Data abstraction of bisacodyl.

**Author, Year Study Design Setting**	**Population Characteristics**		**Interventions**	**Outcomes**	**Relevant Results**
	**SCI**	**Control**			

Amir, 1998<br><br>Prospective Cohort (crossover)<br><br>Bronx VA Medical Center, NY, USA	**N:** 7<br>**Level:** c4-T12; 6 tetraplegics, 1 paraplegic<br>**Etiology:** traumatic **Age:** mean 45 y, range 21–76 y<br>**Duration:** 16 y, range 2–35 y<br>**% Female:** 0%	None	**Intervention:** one w of using bisacodyl suppositories (2 per sessions), glycerin suppositories (2 per sessions), mineral oil enema (1 per session), or Therevac-SB (1 per session) with a 1 w wash out period between each intervention type.<br>**Comparing:** bisacodyl suppositories vs. glycerin suppositories vs. mineral oil vs. Therevac-SB	**Source:** clinical examination<br><br>**Outcomes:** frequency of difficulty evacuating and total and segmental colonic transit time (20 radio-opaque markers administered on three successive days then plain abdominal X-ray taken)	1. Total colonic transit time (colonic transit time) was shortest with Therevac (mean 32 h) compared to Mineral oil enema (34.5 h), bisacodyl suppositories (47.6 h) and glycerin suppositories (48.0 h).<br>2. No significant difference in total colonic transit time between Therevac and mineral oil enema. Both had a shorter colonic transit time than bisacodyl or glycerin suppositories ($p < 0.05$).<br>3. Bowel evacuation time was Therevac (31.5 min), mineral oil (46.5 min), glycerin (57.6 min) and bisacodyl (63.5 min).<br>4. In terms of difficulty with evacuation, Therevac scored best in symptom reduction followed by, in descending order of efficacy, mineral oil, bisacodyl and glycerin.
Stiens, 1998<br><br>Controlled Crossover<br><br>Tampa VA Medical Center SCI Unit, USA	**N:** 14<br>**Level:** c3-L1; 10 complete, 4 incomplete<br>**Age:** mean 53.4 y<br>**Duration:** mean 18.3 y, range >1 y<br>**% Female:** 0%	Control defined as HVB (standard of care).<br><br>Same Population	**Intervention:** 6 consecutive sessions with use of either HVB or PGB<br><br>**Comparing:** HVB sessions vs. PGB sessions<br>**HVB Complications:** incontinence (15 episodes)<br><br>**PGB Complications:** incontinence (1 episodes)	**Source:** in a clinical setting a nurse inserted the medication and recorded outcome measures.<br><br>**Outcomes:** time to flatus, flatus to stool flow, defecation period, clean up time, total bowel care time	1. Time to flatus was shorter for PGB than HVB (12.8 min vs. 31 min; $p < 0.002$).<br>2. Defecation period was shorter for PGB than HVB (32 vs. 58 min; $p < 0.0005$).<br>3. Total bowel care time was shorter for PGB than HVB (51.2 vs. 102 min; $p < 0.0005$).<br>4. The numbers of digital stimulations required for the bowel care sessions was lower for PGB than HVB (3.2 vs. 5; $p < 0.0005$).<br>5. Clean up time and amount of stool produced was not statistically different between interventions.

Frisbie 1997<br><br>Prospective Controlled Trial (cross over)<br><br>Department of VA Medical Center, Brockton, USA	**N:** 19<br>**Level:** 15 cervical, 4 thoracic; all upper motor lesions; 15 motor complete **Etiology:** myelopathy (unspecified)<br>**Age:** mean 64 y, range 41–81 y<br>**Duration:** mean 19 y, range 3–51 y	Control classified as HVB bisacodyl suppositories (standard of care)<br><br>Same population	**Intervention:** 1–2 w of HVB bisacodyl suppositories (10 mg) use or 2 w of PGB bisacodyl suppositories (10 mg)<br><br>**Comparing:** HVB (57 total) vs. PGB (114 total)<br><br>**PGB Complications:** sweating, cramping or delayed fecal incontinence (3 patients), rectal burning (2 patients)<br><br>**HVB Complications:** rectal burning (2 patients; same ones as above).	**Timeline:**<br>Follow-up 3 y later<br><br>**Outcomes:** duration of defecation	1. All patients experienced a shorter bowel care time with PGB. Average time for bowel evacuation was 2.4 hours (range 1.0–4.5 hours) with HVB and 1.1 hours (range 0.3 to 1.8 hours) with PGB.

House and Stiens 1997<br><br>Prospective Randomized Study (crossover, Double-Blind)<br><br><br>USA	**N:** 15<br>**Level:** 9 cervical, 6 thoracic; 11 complete, 4 incomplete<br>**Age:** mean 45 y, range 26–61 y<br>**Duration:** 3 mo to 45 y	None	**Intervention:** 3 sessions with hydrogenated vegetable-oil base bisacodyl suppository (HVB; 10 mg) or polyethylene glycol base bisacodyl suppository (PGB; 10 mg) or Theravac SB (TVC). Drug randomly assigned prior to each regularly scheduled bowel care session.<br><br>**Comparing:** HVB sessions vs. PGB sessions vs. TVC sessions (only given to 10 participants who use it normally)<br><br>**HVB Complications:** incontinence (5 episodes)<br><br>**PGB Complications:** incontinence (3 episodes)<br><br>**TVC Complications:** incontinence (2 episodes)	**Source:** in a clinical setting a blinded nurse inserted the medication and recorded outcome measures.<br><br>**Outcomes:** time to flatus, flatus to stool flow, duration of defecation, incontinence following bowel session, digital rectal stimulation required.	1. Mean time to flatus: PGB less time than HVB (15 vs. 32 min; $p < 0.026$), but similar to results to TVC (15 min).<br>2. Mean duration of defecation: PGB less time than HVB (20 vs. 36 min, $p < 0.037$), but similar to TVC time (17 min).<br>3. Mean total time for bowel program: PGB decreased bowel care time compared to HVB (43 vs. 74.5 min; $p < 0.010$), but similar results to TVC (37 min).<br>4. No significant difference across groups for amount of stool produced, and DRS required.

Dunn and Galka 1994<br><br>Case Series<br><br>San Diego Veterans Affairs Medical Center SCI Center, USA	**N:** 14 (10 completed all treatment phases)<br>**Level:** c5-L1; 5 tetraplegics, 9 paraplegics<br>**Age:** mean 46 y, range 27–67 y<br>**Duration:** mean 19 y range 2–38 y<br>**% Female:** 7.1%	Run in phase with bisacodyl suppositories used to establish a baseline across patients.	**Intervention:** bisacodyl suppositories or TVC enema<br><br>**Comparing:** TVC vs. bisacodyl suppositories<br><br>**TVC complications:** bowel cramping (1 patient)<br><br>**Reasons for withdrawals (Total 4):** TVC ineffective (2 patients), bowel cramping (1 patient) and withdrew consent (1 patient)	**Timeline:** 1992–1993<br>Drug Schedule:<br>Phase 1—Bisacodyl for 5 bowel programs Phase 2—Theravac-SB for 5 bowl programs<br>Phase 3—Bisacodyl suppositories for 5 bowel programs<br><br>**Source:** patient diary log<br><br>**Outcomes:** time of insertion of the rectal medication, time of first evacuation, time required to complete the first evacuation, other interventions used and bowel problems between bowel programs	1. Mean evacuation time was reduced with Theravac SB compared to bisacodyl intervention (phase 1 and 3) ($p < .001$ using MANOVA test; $p < 0.01$ Tukey's post hoc test).

Coggrave, 2009<br><br>Cross-sectional<br><br>National Spinal Injuries Center, UK	**N:** 1334 respondents<br>**Level:** 541 cervical (218 complete, 289 incomplete, 34 unknown), 669 thoracic (399 complete, 207 incomplete, 63 unknown); 112 lumbar (23 complete, 70 incomplete, 19 unknown), 4 sacral (1 incomplete, 3 unknown)<br>**Etiology:** traumatic or acute onset disease<br>**Age:** median 51.5 y, range 19–91 y<br>**Duration:** median 17.6 y, range 1–65 y<br>**% Female:** 26.6%	None	**Objective:** describe bowel management in community-dwelling SCI individuals and to explore associations between age, injury, dependency, problems, interventions, and satisfaction	**Source:** mail-in questionnaire<br><br>**Outcome:** method of evacuation, number of interventions used before finding a successful protocol, bowel care problems and assistance with bowel care	Dulcolax Suppositories related results:<br>1. Associated with the following in cervical injuries (C), thoracic injuries (T) and lumbar/sacral (L/S):<br>a. hemorrhoids C ($p = 0.02$, OR 1.7)<br>b. fecal incontinence ($p = 0.01$)<br>c. abdominal pain ($p = 0.002$)<br>d. longer duration ($p = \leq 0.001$)<br>e. AD ($p = < 0.001$)

Table 5aii. Data abstraction of reviews pertaining to bisacodyl.

Author, Year	Review Design	Aim	Characteristics of Articles	Relevant Results
Yi, 2016	Meta-Analysis<br><br>Bisacodyl Suppositories	To compare the efficacy of polyethylene glycol based (PGB) and vegetable oil based (HVB) bisacodyl suppositories	**N:** 3<br><br>**Types:** 1 prospective Randomized study, 1 prospective controlled study, 1 controlled crossover	1. PGB provided faster outcomes for patients compared to HVB with shorter total bowel care time ($p < 0.05$), time to flatus ($p < 0.05$), and defecation period ($p < 0.05$).

Table 5. b. Grading of evidence—Bisacodyl.

# Studies (design)	Risk of Bias	Inconsistency	Indirectness	Imprecision	Point Estimate(s)	Quality of Evidence Rating (GRADE)
4 controlled crossover and 2 observational, 1 meta-analysis	Serious (only 2 of the controlled trials mentioned randomization and crossover details were lacking)	Serious *formulation unclear in 2 studies	Not serious	Not serious	PGB Bisacodyl<br>Time to flatus: 12.8–15 min<br>Defecation time: 20–32 min<br>Total bowel care time: 43–66 min<br><br>HVB Bisacodyl<br>Time to flatus: 31–32 min<br>Defecation time: 36–58 min<br>Total bowel care time: 74.5–144 min	Low

Table 5ci. Risk of bias for studies of bisacodyl.

Author, Year	Non-biased selection?	Attrition rate below 15%?	Outcomes prespecified and defined?	Methodology adequately described?	Non-biased and adequate methodology?	Statistical analysis of potential confounds?	Adequate duration of follow-up?	Risk of Bias
Coggrave, 2009	No *45.1% response rate	Yes	Yes	Yes	Yes	No	N/A	High *45.1% response rate
Amir, 1998	Unclear	Yes	Yes	No *unclear who accessors are	Unclear	No	N/A	High
Stiens, 1998	Yes	Yes	Yes	Yes	Yes	No	N/A	Moderate

Frisbie, 1997	Unclear	Yes	Yes	Yes	No *interventions not compared for equal duration	No	No *follow-up after 3 y following a 3–4 w program	High
House and Stiens, 1997	Yes	Yes	Yes	Yes	Yes	No	N/A	Moderate
Dunn and Galka, 1994	Yes	No *18.6% attrition rate	Yes	Yes	No *significance report, but not mean values	No	N/a	High *Missing data

Table 5cii. Risk of bias of review studies of bisacodyl.

**Author, Year**	**Report clear review question, state inclusion and exclusion of primary studies?**	**Substantial effort to find relevant research?**	**Adequate assessment of validity of included studies?**	**Sufficient detail of individual studies presented?**	**Primary studies summarized appropriately?**
Yi, 2016	Yes	Yes	No	Yes	Yes

Table 6a. Data abstraction of fampridine and SCI.

**Author, Year Study Design Setting**	**Population Characteristics**		**Interventions**	**Outcomes**	**Results**
	**SCI**	**Control**			
Cardenas, 2014<br><br>RCT # 1 (double-blind, multicenter, phase III)	**Trial 1**<br>**N:** 114 (114 analyzed)<br>**Level:** all incomplete; AIS: 42 B, 29 C, 43 D<br>**Etiology:** 49 vehicle accident, 23 fall, 10 diving, 8 sports, 9	Control defined as a placebo.<br><br>**N:** 99 (98 analyzed)	**Interventions:** 25 mg fampridine-SR (slow release) or placebo twice daily<br><br>**Comparing:** placebo vs. experimental, before vs. after<br><br>**Reasons for withdrawals (46 total):**	20 w protocol- 2 w placebo period, 2 w dose titration phase, 12 w at fixed target dose, 2 w downward titration, and 2 w washout.	1. No significant difference between treatment groups for subject global impression.<br>2. No significance difference

North America	gunshot wound, 15 other<br>**Age:** mean 41.6 y, SD 12.1 y<br>**Duration:** ≥ 18 mo<br>**% Female:** 12.3%	**Level:** all incomplete; AIS: 28 B, 32C, 38 D<br>**Etiology:** 48 vehicle accident, 13 fall, 13 diving, 8 sports, 7 gunshot wound, 9 other<br>**Age:** mean 40.1 y, SD 13.1 y<br>**Duration:** ≥ 18 mo<br>**% Female:** 13.3%	noncompliance (5 fampridine vs. 6 placebo), adverse events (20 fampridine vs. 2 placebo), request by subject (3 fampridine vs. 1 placebo), lost to follow-up (0 fampridine vs. 2 placebo), other (5 fampridine vs. 2 placebo)<br><br>**Complications** (more frequent in fampridine): UTI's, dizziness, constipation, headache, nausea, paresthesia, insomnia, asthenia, back pain, dyspepsia, nervousness, rash, sweating, anxiety, urinary incontinence, abdominal pain, peripheral edema, fever	**Source:** physical assessments, laboratory evaluations, self-reports, questionnaires, staff observations, neurological examination<br><br>**Relevant outcomes:** duration and frequency of bowel movements, and subject global impression (SGI)	between treatment groups for bowel outcomes<br>3. Most Common Adverse Events related to Withdrawal were dizziness (6.1%) and hypertonia, insomnia, and asthenia (2.6% each)
Cardenas, 2014<br><br>RCT # 2 (Double-Blind, Multicenter, Phase III)<br><br>North America	**Trial 2**<br>**N:** 104(103 analyzed)<br>**Level:** all incomplete; AIS: 32 B, 26 C, 45 D<br>**Etiology:** 42 vehicle accident, 16 fall, 16 diving, 7 sports, 4 gunshot wound, 16 other<br>**Age:** mean 41.3 y, SD 11.8 y<br>**Duration:** ≥ 18 mo<br>**% Female:** 14.0%	Control defined as a placebo.<br><br>**N:** 100 (100 analyzed)<br>**Level:** all incomplete; AIS: 32 B, 30 C, 38 D<br>**Etiology:** 40 vehicle accident, 18 fall, 16 diving, 13 sports, 7 gunshot wound, 8 other<br>**Age:** mean 40.5 y, SD 12.3 y<br>**Duration:** ≥ 18 mo	**Interventions:** 25 mg fampridine-SR (Slow Release) or placebo twice daily<br><br>**Comparing:** placebo vs. experimental, before vs. after<br><br>**Reasons for withdrawals (38 total):** noncompliance (3 fampridine vs.1 placebo), adverse events (12 fampridine vs. 2 placebo), request by subject (6 fampridine vs. 5 placebo), lost to follow-up (1 fampridine vs. 1 placebo), other (4 fampridine vs. 3 placebo)<br><br>**Complications** (more frequent in fampridine): UTIs, hypertonia, dizziness, pain, constipation, headache, nausea, paresthesia,	**Timeline:** Jun 2002–Nov 2003.<br><br>20 w protocol—2 w placebo period, 2 w dose titration phase, 12 w at fixed target dose, 2 w downward titration, and 2 w washout.<br><br>**Source:** physical assessments, laboratory evaluations, self-reports, questionnaires, staff observations, neurological examination<br><br>**Relevant outcomes:** duration and frequency of	1. No significant difference between treatment groups for subject global impression.<br>2. Greater increase with fampridine-SR relative to placebo for the number of bowel movements ($p < 0.006$).<br>3. Most Common Adverse Events related to Withdrawal were dizziness (3.9%), hypertonia (2.9%) and paresthesia (2.9%)

		**% Female:** 16.5%	insomnia, back pain, nervousness, arthralgia, sweating, anxiety, abdominal pain, fever	bowel movements, and subject global impression (SGI)	
Cardenas, 2007<br><br>RCT (double-blind)<br><br>USA	**Fampridine-SR 25 mg N:** 30 (26 completed)<br>**Level:** 23 cervical, 7 thoracic; AIS: 14 C, 16 D<br>**Age:** mean 44 y range 23–66 y<br>**Duration:** mean 8.3 y range 1–30 y<br>**% Female:** 27%<br><br>**Fampridine-SR 40 mg**<br>**N:** 30 (17 completed)<br>**Level:** 24 cervical, 6 thoracic; AIS: 12 C, 18 D<br>**Age:** mean 42 y range 21–67 y<br>**Duration:** mean 10.8 y range 1–35 y<br>**% Female:** 4%	Control defined as a placebo with same composition as study drug with no active ingredients.<br>Placebo:<br>**N:** 31 (28 completed)<br>**Gender:** 24 M 7 F<br>**Age:** mean 38 y range 19–61<br>**Level:** 26 cervical, 5 thoracic; AIS: 18 C, 13 D<br>**Duration:** mean 8.3 y, range 1–37 y	**Interventions:** 25 mg BID fampridine-SR (Slow Release) or 40 mg BID fampridine-SR<br><br>**Comparison Groups:** placebo vs. 25 mg BID fampridine-SR vs. 40 mg BID fampridine-SR<br><br>**Reasons for withdrawals (14 total):**<br>- Es contributed to 10 withdrawals; most common associated AEs were dizziness (8%), insomnia (4%) and nausea (3%)<br>- 2 patients lost to follow-up and 2 withdrew due to undisclosed reasons.	**Timeline:** June 27, 2000 to March 6, 2001.<br><br>11 w protocol—2 w placebo period, 2 w dose titration phase, 4 w at fixed target dose, 2 w downward titration, and 1 w washout.<br><br>**Source:** physical assessments, laboratory evaluations, self-reports, questionnaires, staff observations, neurological examination<br><br>**Outcomes:** frequency of bowel movements, and subject global impression (SGI)	1. Overall positive response rates on PDQ were low and not significantly different between treatment groups.<br>2. 19% of the 25 mg BID group and 23% of the 40 mg BID group had an increase in number of days with bowel movements vs. 0% of the placebo group (p = 0.02 and p = 0.01, respectively).<br>3. SGI mean was higher in subjects taking 25 mg BID compared to placebo (4.5 and 3.9, respectively. p = 0.02).
Potter, 1998<br><br>RCT (Double-Blind, Crossover)<br><br>North America	**N:** 29 (26 complete)<br>**Level:** 19 tetraplegic, 10 paraplegic; all incomplete; ASI Score: 6 B, 12 C, 11 D<br>**Age:** mean 40.6 y, SD 10.0 y<br>**Duration:** mean 152 mo, SD 105.4 mo	Crossover with 1 w wash out period in-between.	**Interventions:** fampridine-SR (Slow Release) or placebo twice daily<br><br>**Comparing:** placebo data vs. experimental data, before vs. after<br><br>**Reasons for withdrawals (3 total):**	**Fampridine Protocol:**<br>1 w of 12.5 mg BID, and 1 w of 17.5 mg BID<br><br>**Source:** clinical assessment and questionnaires	1. No significant difference between treatment groups for sphincter control<br>2. Under fampridine treatment participants reported a higher quality of life (p = 0.011)

	**% Female:** 3.4%		severe respiratory and urinary tract infections (2 participants), undisclosed reason (1 participant)<br><br>**Fampridine Complications:** mild and transient giddiness or lightheaded (5 participants), severe respiratory and urinary tract infections (2 participants)	**Relevant outcomes:** QoL and Functional Independence Measure (Sphincter Control)	3. There were no statistically significant benefits of the drug on measures of pain or bowel, bladder and sexual function, or functional independence.

Table 6b. Grading of body of evidence—fampridine.

# Studies (design)	Risk of Bias	Inconsistency	Indirectness	Imprecision	Point Estimate(s)	Quality of Evidence Rating (GRADE)
4 (RCT)	Serious<br>*unclear group assignment	Serious<br>*cannot pool data; dosing varied	Not serious	Serious<br>*less than 400 sample size	12.5 mg and 17 mg—improved quality of life (p = 0.011).<br><br>25 mg BID—increased number of days with bowel movements (in 2/3 RCTs).<br><br>40 mg BID—increased number of days with bowel movements (23% of group vs. 0% of placebo group; p = 0.02).	Low

Table 6c. Risk of bias—fampridine.

Author, Year	Randomization adequate?	Allocation concealment adequate?	Groups similar at baseline?	Eligibility criteria specified?	Participants and personnel blinded?	Outcome assessors blinded?	No evidence of unreported outcomes?	Attrition rate below 15%?	Analyzed according to randomization?	Risk of Bias
Cardenas, 2014 (#1 and 2)	Unclear	Unclear	Yes	Yes	Yes	Unclear	Yes	Yes	Yes	Moderate

Cardenas, 2007	Unclear	Unclear	Yes	Yes	Yes	Yes	Unclear	No *43% of 40 mg BID withdrew	Yes	High
Potter, 1998	Unclear	Unclear	Yes	Yes	Yes	Unclear	Unclear	Yes	Unclear	High *Unclear design

Table 7a. Fampridine on NBD management for MS.

Author, Year<br>Study Design<br>Setting	Population Characteristics		Interventions	Outcomes	Results
	**Drug Intervention**	**Control**			
Polman et al. 1994<br><br>Case series study<br><br>University referral center	<u>**1st subgroup:**</u><br>**N:** 12<br>**Age:** 42.7 y<br>**Duration of MS:** n/a<br>**% Female:** 6<br><br><u>**2nd subgroup:**</u><br>**N:** 19<br>**Age:** 48.4 y<br>**Duration of MS:** 3–27 y<br>**% Female:** 11		**Intervention:**<br>Long-term oral treatment with 4-aminopyridine in daily doses of up to 0.5 mg/kg of body weight.<br><br>1st Subgroup: patients who had participated in a 4-ap study took their previously preferred dose.<br>2nd Subgroup: patients who had not been treated previously started with a dose of 10 to 15 mg/d in 2–3 divided doses, then elevated in 4 to 8 w up to a maximum dose of 0.5 mg/kg of body weight.<br><br>**Objective:**<br>to examine the incidence, severity, and duration of GI-related events in DMF patients and to describe the impact of symptomatic therapy.	**Time:**<br>All patients visited the outpatient department at regular (usually 3-mo) intervals.<br>**Source:**<br>Neurologic symptoms, functions, side effects, concomitant diseases, other medications used reported by patients.<br><br>**Outcomes:**<br>neurologic functions and symptoms as reported by the patients including side effects.	1. 1 out of 23 patients reported subjective improvements in urinary/fecal incontinence after continuing 4-aminopyridine therapy for more than 6 mo.<br>2. 18 patients reported subjective improvement in ability to perform the activities of normal daily life, which was mainly owing to improved ambulation and reduced fatigue.

Table 7b. Laxatives and suppositories on NBD management for MS.

**Author, Year Study Design Setting**	**Population Characteristics**	**Interventions**	**Outcomes**	**Results**
Norton and Chelvanayagam, 2010<br><br>Survey/Questionnaire<br><br>UK	<u>Results from online survey:</u><br><br>N: 155<br><br>Age: median-50 y, range-19–73 y<br><br>Duration of MS: n/a<br><br>% Female: 77%<br><br><u>Results from posted questionnaire:</u><br><br>N: 47<br><br>Age: 58 y (28–76 y)<br><br>Duration of MS: the start of MS symptoms range: 2–28 y (mean 20 y)<br><br>% Female: 93.6%	**Methods:**<br><br>Conducted a 2 stage survey: an initial brief online survey and a more comprehensive questionnaire. The invitation to participate in the survey was specifically aimed towards people with MS.<br><br>**Objective:**<br><br>This survey aimed to describe the impact bowel dysfunction has on lives of people with MS, and to identify interventions that are helpful/may warrant further investigation.	**Time:** n/A<br><br>**Outcomes:**<br><br>The effect of respondents' bowel symptoms on their everyday life.<br><br>Bowel management methods for constipation/evacuation difficulties, for fecal incontinence, bowel management and its effect on lifestyle, time spent on bowel management daily.	<u>Survey Results:</u><br><br>1. More than 60% reported constipation and 30% fecal incontinence, with nearly 20% experiencing both.<br><br>2. Medication was found to be the most helpful intervention for managing fecal incontinence and constipation and people were prescribed a range of medication for both bladder and bowel dysfunction.<br><br>3. Laxatives were the most commonly used bowel management method for constipation (84 respondents reported using—12 said "not at all helpful", 22 said "slightly helpful", 29 said "moderately helpful", 21 said "very helpful".<br><br>4. 38 respondents reported using suppositories—8 said "not at all helpful", 8 said "slightly helpful", 9 said "moderately helpful", and 13 said "very helpful".<br><br>5. In comments, participants reported that fecal incontinence has a more profound effect on quality of life than constipation.

Table 7c. Grading of body of evidence on MS papers.

# Studies (design)	Risk of Bias	Inconsistency		Indirectness	Imprecision	Point Estimate(s)	Quality of Evidence Rating (GRADE)
2 (Obs)	Serious *Survey convenience sampling	Not serious		Very serious *Self-report of bowel dysfunction only	Serious *Less than 400 participants	60% reported constipation (1 study) and 5–30% reported fecal incontinence (2 studies)	Very low

Table 7d. Risk of bias assessment for MS papers.

Author, Year	Non-biased selection?	Attrition rate below 15%?	Outcomes prespecified and defined?	Methodology adequately described?	Non-biased and adequate methodology?	Statistical analysis of potential confounds?	Adequate duration of follow-up?	Risk of Bias
Polman, 1994	Unclear *follow-up with MS patients at specific health center	Yes	No *as reported by patients	Yes	Yes	No	N/A	High *direct survey only
Norton, 2010	No *subscribers to MS newsletter opted into survey	Unclear	Yes	Yes	Yes	No	N/A	High *unclear selection criteria for online survey

Table 8. Studies on medication/laxatives and SCI (general).

Author, Year Study Design Setting	Population Characteristics	Interventions	Outcomes	Results
Ozisler et al. 2015<br><br>Survey	N = 42 (76%) males and 13 (24%) females.	None.<br><br>Objective: to determine GI problems associated with NBD in SCI	At the beginning and end of bowel program, medications (oral laxatives, suppositories, enemas) and bowel	1. Constipation (56%, 31/55) and incontinence (42%, 23/55) were the most common gastrointestinal problems.

Turkey	Mean age = 33.01 ± 12.25 y.<br><br>Mean interval since injury = 162.0 ± 110.1 (21–360) days<br><br>37 (67%) patients had motor complete SCI and 18 (33%) patients had motor incomplete SCI.	patients and to assess the efficacy of bowel program on GI problems and the severity of NBD.	evacuation methods (digital stimulation, abdominal massage, enema, Valsalva maneuver and manual evacuation) for bowel care used by patients were recorded.<br><br>NBD scores of patients were calculated.	2. The mean NBD score in patients with motor complete SCI was significantly higher than in patients with motor incomplete SCI before (17.45 ± 6.37 vs. 8.44 ± 9.39, P = 0.001) and after (11.40 ± 3.58 vs. 5.22 ± 6.38; P = 0.000) bowel program.<br><br>3. After bowel program, the mean NBD score was significantly decreased in both patients with motor complete (P = 0.000) and incomplete (P = 0.018) SCI patients. After bowel program, the NBD score was significantly reduced in patients with motor complete SCI than in patients with motor incomplete SCI (6.05 ± 4.66 vs. 3.27 ± 4.65; P = 0.017).<br><br>4. Oral medication (P = 0.016), enema (P = 0.001) and manual evacuation (P = 0.008) application rates significantly decreased at the end of bowel program when compared to the beginning of bowel program.
De Looze et al. 1998<br><br>Survey<br><br>Belgium	N= 78 (63 men; 15 women)<br><br>Median age of 37.5 y (range 18 ± 72 y).<br><br>Median duration of injury = 7 y (range 5 mo ± 22 y).<br><br>Cause of injury—traumatic = 71; medical = 7<br><br>23 tetraplegic and 55 paraplegic patients (32 with lesions above T10, 23 T10 and lower). The lowest level of injury was L2.	None.	Survey asked about medications taken and presence of fecal incontinence, constipation, and other GI problems.	1. Anticholinergic drug intake was found to be significantly related to constipation (P=0.03). Anticholinergics and muscle relaxants were used by 32 and 30 patients, respectively.<br><br>2. 58% of patients with a complete SCI above L2 suffered from constipation. Tetraplegic patients had the highest prevalence of constipation, while patients with low paraplegia were less prone to constipation.<br><br>3. 15 patients used laxatives on a regular basis.<br><br>4. Thirty-seven patients had never experienced fecal incontinence. Occasional fecal incontinence was mentioned by 38 patients and daily fecal incontinence by 3 patients.

				5. There was no statistically significant relationship between the presence of constipation and fecal incontinence.
Harari et al. 1997<br><br>Cross-sectional study<br><br>USA	N = 161<br><br>Mean age = 57+15 y (97% male).<br><br>Mean y since injury = 20.4+14.<br><br>Cervical 56%, thoracic 35%, lumbosacral 9%.<br><br>An average of 5.9+4.0 medications were taken per participant.	None.<br><br>Objective: to investigate possible associations between bowel dysfunction and age, sex, level of injury, time since injury, health status, and SCI.	Outcomes: incontinence occurring at least once per mo (self-report), current regular laxative use (four doses per mo), laxative use prior to SCI, current use of at least six medications (calcium channel blockers, iron supplements, antidepressants, neuroleptics, baclofen, narcotics, antidiarrheals, and nonsteroidal anti-inflammatory drugs).<br><br>Chi-squared tests were used to examine differences in demographic, clinical and pharmacological characteristics between interview responders and nonparticipants.	1. After inclusion in a multiple logistic regression model, the factors that were independently associated with difficulty with evacuation were: tetraplegia (Adjusted Odds Ratio (AOR) 2.79 95% CI 1.3 ± 6.2), Frankel grade A or B (AOR 2.77 95% CI 1.1 ± 6.7), four laxative doses taken per mo (AOR 2.41 95% CI 1.0 ± 6.6), and long-term care residency (AOR 0.21 95% CI 0.1 ± 0.6).<br><br>2. 69% of participants reported use of at least four laxative, suppository, or enema doses per mo, at an average number of 27+38 doses per mo.<br><br>3. The most popular oral agent, docusate sodium was used by 21% of participants. Stimulant laxatives (bisacodyl, senna and cascara) were the second most commonly used agents (16%). 41% of residents reported use of bisacodyl suppositories, and 6% used enemas.<br><br>4. One-third of individuals described at least one episode of fecal incontinence a mo, and 22% felt that evacuation was facilitated following a meal or a drink.
Han et al. 1998<br><br>Interview study<br><br>South Korea	Seventy-two patients voluntarily participated in this interview study (all were from one national university hospital); 48 males and 24 females.<br><br>Average age = 38.0+11.9 y.	None	Semi-structured individual Interviews included questions relating to G-I problems significant enough to have adverse impact on ADLs or require long-term management.	1. Age, duration, level, ASIA score, and ADL status were not related to bowel dysfunction.<br><br>2. 43% of the patients took oral medication and 36.1% controlled their diet to improve bowel function.<br><br>3. Severe constipation was the most common problem, affecting 43.1% of patients, while difficulty with evacuation (33.3%) and post-

	Mean duration of spinal cord injury = 37.5+45.2 mo.<br><br>**Level of Injury:** c4-C8 = 26; T1-T6 = 20; Below T6 = 26<br><br>ASIA Score = A = 32; B = 15; C = 20; D = 5		Daily activity level (ADL) was evaluated using the Modified Barthel Index score, and patients were grouped according to their level of dependence (total, severe, moderate, or independent).<br><br>Chi-squared tests were used to determine the association between age, duration, level, ASIA score, ADL status, and bowel dysfunction.	prandial discomfort (33.3%) were the next most frequent.

Table 8b. Grading of body of evidence—general SCI and medication studies.

# Studies (design)	Risk of Bias	Inconsistency	Indirectness	Imprecision	Point Estimate(s)	Quality of Evidence Rating (GRADE)
4 (Obs)	Serious<br>*all convenience sampled from one center	Very serious<br>*Different outcomes measured, cannot pool datal	Serious<br>*self-report and interview data	Serious<br>*Less than 400 participants	Constipation was experienced by 40–58% of participants (3 studies) and fecal incontinence by 13–43% of participants (4 studies). Oral medications were used by 36–69% of participants (4 studies) and suppositories by 15–89% of participants (2 studies).	Very low

Table 8c. Risk of bias for observational studies of medications and suppositories—general in SCI.

Author, Year	Non-biased selection?	Attrition rate below 15%?	Outcomes prespecified and defined?	Methodology adequately described?	Non-biased and adequate methodology?	Statistical analysis of potential confounds?	Adequate duration of follow-up?	Risk of Bias

Ozisler, 2015	No *patients all with SCI from one rehab center	Yes	Yes	Yes	Yes	Yes *specific nonparametric tests used where appropriate	N/A	Moderate
De Looze, 1998	Unclear * randomization, but sampling was from one health center only	Yes	Yes	Yes	Yes	No	Yes	Moderate
Harari, 1997	No *patients all with SCI from one rehab center	Yes	Yes	Yes	Yes	No	N/A	High
Han, 1998	No *patients all with SCI from one rehab center	Yes	Yes	Yes	Yes	No	N/A	High

Included Studies

1. Krogh K, Jensen MB, Gandrup P, Laurberg S, Nilsson J, Kerstens R, De Pauw M. Efficacy and tolerability of Prucalopride in patients with constipation due to spinal cord injury. *Scand J Gastroenterol*. 2002, *37*, 431-436. [CrossRef][PubMed]

2. Segal, J.L.; Milne, N.; Brunnemann, S.R.; Lyons, K.P. Metoclopramide-induced normalization of impaired gastric emptying in spinal cord injury. *Am J Gastroenterol*. 1987, *82*, 1143-1148. [PubMed]

3. Miller, F.; Fenzl, T.C. Prolonged ileus with acute spinal cord injury responding to metaclopramide. *Paraplegia*. 1981, *19*, 43-45. [CrossRef][PubMed]

4. Rosman, A.S.; Chaparala, G.; Monga, A.; Spungen, A.M.; Bauman, W.A.; Korsten, M.A. Intramuscular neostigmine and glycopyrrolate safely accelerated bowel evacuation in patients with spinal cord injury and defecatory disorders. *Dig Dis Sci*. 2008, *53*, 2710-2713. [CrossRef][PubMed]

5. Korsten, M.A.; Rosman, A.S.; Ng, A.; Cavusoglu, E.; Spungen, A.M.; Radulovic, M.; Wecht, J.; Bauman, W.A. Infusion of neostigmine–glycopyrrolate for bowel evacuation in persons with spinal cord injury. *Am J Gastroenterol*. 2005, *100*, 1560-5. [PubMed]

6. Korsten, M.A.; Lyons, B.L.; Radulovic, M.; Cummings, T.M.; Sikka, G.; Singh, K.; Hobson, J.C.; Sabiev, A.; Spungen, A.M.; Bauman, W.A. Delivery of neostigmine and glycopyrrolate by iontophoresis: a nonrandomized study in individuals with spinal cord injury. *Spinal Cord*. 2018, *56*, 212-217. [CrossRef][PubMed]

7. Hwang, M.; Zebracki, K.; Vogel, L.C. Long-term outcomes and longitudinal changes of neurogenic bowel management in adults with pediatric-onset spinal cord injury. *Arch Phys Med Rehabil.* 2017, *98*, 241-248.[CrossRef][PubMed]

8. Furusawa, K.; Sugiyama, H.; Tokuhiro, A.; Takahashi, M.; Nakamura, T.; Tajima, F. Topical anesthesia blunts the pressor response induced by bowel manipulation in subjects with cervical spinal cord injury. *Spinal Cord.* 2009, *47*, 144-148. [CrossRef][PubMed]

9. Furusawa, K.; Tokuhiro, A.; Sugiyama, H.; Tajima, F.; Genda, E.; Uchida, R.; Tominaga, T.; Tanaka, H.; Magara, A.; Sumida, M. Incidence of symptomatic autonomic dysreflexia varies according to the bowel and bladder management techniques in patients with spinal cord injury. *Spinal Cord.* 2011, *49*, 49-54. [CrossRef][PubMed]

10. Furusawa, K.; Sugiyama, H.; Ikeda, A.; Tokuhiro, A.; Koyoshi, H.; Takahashi, M.; Tajima, F. Autonomic dysreflexia during a bowel program in patients with cervical spinal cord injury. *Acta Med Okayama*. 2007, *61*, 211-227. [CrossRef][PubMed}

11. Inskip, J.A.; Lucci, V.M.; McGrath, M.S.; Willms, R.; Claydon, V.E. A community perspective on bowel management and quality of life after spinal cord injury: The influence of autonomic dysreflexia. *J Neurotrauma*. 2018, *35*, 191-1105. [CrossRef][PubMed]

12. Adriaansen, J.J.; van Asbeck, F.W.; van Kuppevelt, D.; Snoek, G.J.; Post, M.W. Outcomes of neurogenic bowel management in individuals living with a spinal cord injury for at least 10 years. *Arch Phys Med Rehabil*. 2015, *96*, 905-912. [CrossRef][PubMed]

13. Coggrave, M.; Norton, C.; Wilson-Barnett, J. Management of neurogenic bowel dysfunction in the community after spinal cord injury: a postal survey in the United Kingdom. *Spinal Cord*. 2009, *47*, 323-330. [CrossRef][PubMed]

14. Amir, I.; Sharma, R.; Bauman, W.A.; Korsten, M.A. Bowel care for individuals with spinal cord injury: comparison of four approaches. *J Spinal Cord Med*. 1998, *21*, 21-24. [CrossRef][PubMed]

15. Stiens, S.A.; Luttrel, W.; Binard, J.E. Polyethylene glycol versus vegetable oil based bisacodyl suppositories to initiate side-lying bowel care: A clinical trial in persons with spinal cord injury. *Spinal Cord*. 1998, *36*, 777-781. [CrossRef][PubMed]

16. Frisbie, J.H. Improved bowel care with a polyethylene glycol based bisacadyl suppository. *J Spinal Cord Med*. 1997, *20*, 227-229. [CrossRef][PubMed]

17. House, J.G.; Stiens, S.A. Pharmacologically initiated defecation for persons with spinal cord injury: effectiveness of three agents. *Arch Phys Med Rehabil*. 1997, *78*, 1062-1065. [CrossRef][PubMed]

18. Dunn, K.L.; Galka, M.L. A comparison of the effectiveness of Therevac SB and bisacodyl suppositories in SCI patients' bowel programs. *Rehabil.Nurs*. 1994, *19*, 334-338. [CrossRef][PubMed]

19. Yi, Z.; Jie, C.; Wenyi, Z.; Bin, X.; Hongzhu, J. Comparison of efficacies of vegetable oil based and polyethylene glycol based bisacodyl suppositories in treating patients with neurogenic bowel dysfunction after spinal cord injury: a meta-analysis. *Turk J Gastroenterol.* 2014, *25*, 488-492. [CrossRef][PubMed]

20. Cardenas, D.D.; Ditunno, J.F.; Graziani, V.; McLain, A.B.; Lammertse, D.P.; Potter, P.J.; Alexander, M.S.; Cohen, R.; Blight, A.R. Two phase 3, multicenter, randomized, placebo-controlled clinical trials of fampridine-SR for treatment of spasticity in chronic spinal cord injury. *Spinal Cord*. 2014, *52*, 70-76. [CrossRef][PubMed]

21. Cardenas, D.D.; Ditunno, J.; Graziani, V.; Jackson, A.B.; Lammertse, D.; Potter, P.; Sipski, M.; Cohen, R.; Blight, A.R. Phase 2 trial of sustained-release fampridine in chronic spinal cord injury. *Spinal Cord*. 2007, *45*,158-168. [CrossRef][PubMed]

22. Potter, P.J.; Hayes, K.C.; Segal, J.L.; Hsieh, J.T.; Brunnemann, S.R.; Delaney, G.A.; Tierney, D.S.; Mason, D. Randomized double-blind crossover trial of fampridine-SR (sustained release 4-aminopyridine) in patients with incomplete spinal cord injury. *J Neurotrauma*. 1998, *15*, 837-849. [CrossRef][PubMed]

23. Polman, C.H.; Bertelsmann, F.W.; van Loenen, A.C.; Koetsier, J.C. 4-Aminopyridine in the treatment of patients with multiple sclerosis: long-term efficacy and safety. *Arch Neurol*. 1994, *51*, 292-296. [CrossRef][PubMed]

24. Norton, C.; Chelvanayagam, S. Bowel problems and coping strategies in people with multiple sclerosis. *Br J Nurs*. 2010, *19*, 221-6. [CrossRef][PubMed]

25. Ozisler, Z.; Koklu, K.; Ozel, S.; Unsal-Delialioglu, S. Outcomes of bowel program in spinal cord injury patients with neurogenic bowel dysfunction. *Neural Regen Res.* 2015, 10, 1153-1158. [CrossRef] [PubMed]

26. De Looze, D.; Van Laere, M.; De Muynck, M.; Beke, R.; Elewaut, A. Constipation and other chronic gastrointestinal problems in spinal cord injury patients. *Spinal Cord*, 1998, 36, 63-66. [CrossRef] [PubMed]

27. Harari, D.; Sarkarati, M.; Gurwitz, J.H.; McGlinchey-Berroth, G.; Minaker, K.L. Constipation-related symptoms and bowel program concerning individuals with spinal cord injury. *Spinal Cord*. 1997, 35, 394-401. [CrossRef] [PubMed]

28. Han TR, Kim JH, Kwon BS. Chronic gastrointestinal problems and bowel dysfunction in patients with spinal cord injury. Spinal Cord 1998; 36: 485-490. [CrossRef] [PubMed]

29.
